# Cryptic variation in RNA-directed DNA-methylation controls lateral root development when auxin signalling is perturbed

**DOI:** 10.1038/s41467-019-13927-3

**Published:** 2020-01-10

**Authors:** Zaigham Shahzad, Ross Eaglesfield, Craig Carr, Anna Amtmann

**Affiliations:** Institute of Molecular, Cell and Systems Biology, College of Medical, Veterinary and Life Sciences, University of Glasgow, Bower Building, Glasgow, G12 8QQ UK

**Keywords:** DNA methylation, Natural variation in plants, Plant morphogenesis

## Abstract

Maintaining the right balance between plasticity and robustness in biological systems is important to allow adaptation while maintaining essential functions. Developmental plasticity of plant root systems has been the subject of intensive research, but the mechanisms underpinning robustness remain unclear. Here, we show that potassium deficiency inhibits lateral root organogenesis by delaying early stages in the formation of lateral root primordia. However, the severity of the symptoms arising from this perturbation varies within a natural population of *Arabidopsis* and is associated with the genetic variation in *CLSY1*, a key component of the RNA-directed DNA-methylation machinery. Mechanistically, *CLSY1* mediates the transcriptional repression of a negative regulator of root branching, *IAA27*, and promotes lateral root development when the auxin-dependent proteolysis pathway fails. Our study identifies DNA-methylation-mediated transcriptional repression as a backup system for post-translational protein degradation which ensures robust development and performance of plants in a challenging environment.

## Abstract (web summary)

Developmental plasticity of plant root systems has been intensively studied, but the mechanisms underpinning robustness remain unclear. Here, the authors show that DNA-methylation-mediated transcriptional repression serves as a backup system to control lateral root development when auxin signalling is perturbed.

## Introduction

Developmental processes are tightly regulated to achieve a balance between plasticity and robustness in response to genetic and environmental perturbation^1,2^. Plasticity of developmental programs is particularly important for immobile organisms, such as plants, because it allows them to optimally explore their immediate environment. For example, plants can adjust the architecture of their root systems in response to edaphic signals, such as mineral nutrient concentrations and water gradients, and thus effectively track soil resources^3–5^. Understanding how individual parts of the root respond to nutrient depletion in the soil is important for selecting root architectures that can support crop yield with minimal fertilizer input.

Number and length of lateral roots (LRs) make an important contribution to shaping root system architecture. LRs emerge from xylem-pole cell triplets, so-called founder cells (FCs), which occur at regular longitudinal intervals in the pericycle layer that surrounds the central vasculature^6^. A local rise in the hormone auxin triggers FCs to undergo several rounds of anticlinal asymmetric divisions that produce a single-layered file of small and larger cells (stage-I LR primordia), followed by a round of periclinal divisions that yield the two-layered stage-II LR primordia. Additional divisions eventually form fully organised root meristems, which penetrate the outer root tissues and generate visible new lateral roots. The transcriptional programmes that underpin the different stages of LR development are activated by auxin responsive factors (ARFs), which bind to auxin responsive elements (AuxRe) in the promoters of their target genes. ARF regulation underlies the same basic steps of auxin signalling that are found in other developmental contexts^6^. In the absence of auxin, ARFs are inhibited by auxin co-receptors called IAAs. When an auxin receptor, e.g. TIR1, binds auxin it recruits the IAA and makes it available for degradation through the ubiquitin proteasome pathway. IAA degradation releases the brake on ARFs thereby inducing the downstream transcriptional changes that lead to LR formation. Given the importance of the auxin signalling pathway for LR development it is not surprising that many environmental signals target this pathway to modulate root architecture, both locally, e.g. by regulating auxin synthesis or transport, and systemically through mobile peptides and cross-talk with other hormonal pathways^5–7^.

Plasticity allows plants to adapt to changing conditions but can also be a disadvantage. For example, inhibiting growth and development in response to unfavourable environmental conditions saves resources but also causes unnecessary delays if problems only occur transiently or in a small area. Therefore, mechanisms to either lower the sensitivity of signalling pathways to environmental cues or over-ride downstream effects on phenotype should also have evolved. At the cellular level there is evidence that molecular buffering mechanisms such as chaperones can provide robustness^2^, but how the conflict between developmental plasticity and robustness is resolved at the population level is not clear. Identifying mechanisms that determine the plasticity/robustness set point of developmental processes is critical for understanding how genetic variation contributes to species performance under environmental change.

Previously, we have shown that potassium (K) deprivation alters LR development in *Arabidopsis thaliana* and that this response differs among *Arabidopsis* accessions^8^. Based on a genome-wide association (GWA) study of lateral root development under low-K conditions, we report here that genetic variation in a CLASSY chromatin remodelling factor (*CLSY1*) determines the robustness of LR development against perturbation of the auxin pathway by low K. *CLSY1* is a component of a RNA-directed DNA methylation (RdDM) complex, which silences a distinct subset of transposable elements (TEs) and genes^9,10^ via a well-characterised molecular machinery^9^. RdDM exhibits a large natural variation in *Arabidopsis*^11^, but the physiological significance of this variation has remained unclear. Using a combination of cell biology and molecular genetics experiments we provide evidence that *CLSY1*-mediated silencing of *IAA27* provides a ‘backup switch’ for maintaining lateral root development when the canonical auxin-mediated pathway is disrupted. Genetic variation in this backup circuit is cryptic and only becomes apparent under environmental or genetic challenge.

## Results

### Low K disturbs early LR development

Previous research in our laboratory revealed that a low concentration of K in the root environment has profound effects on the root system architecture of *Arabidopsis*^8^. In the reference accession Col-0, low K supply (10 µM) decreases both main root (MR) length and LR number compared to control (2 mM), resulting in reduction of total root size compared to control conditions (Supplementary Fig. 1). The decrease in MR length is at least partially due to a decrease of cell length under low K (Supplementary Fig. 2). The low-K induced brake on root growth and development is released when the supply of iron (Fe) in the media is decreased from moderate (control, 42.5 µM) to low (but sufficient, 4.25 µM) levels. Lowering Fe alone slightly increases MR length but has no effect on LR number (Supplementary Fig. 1).

**Fig. 1:**
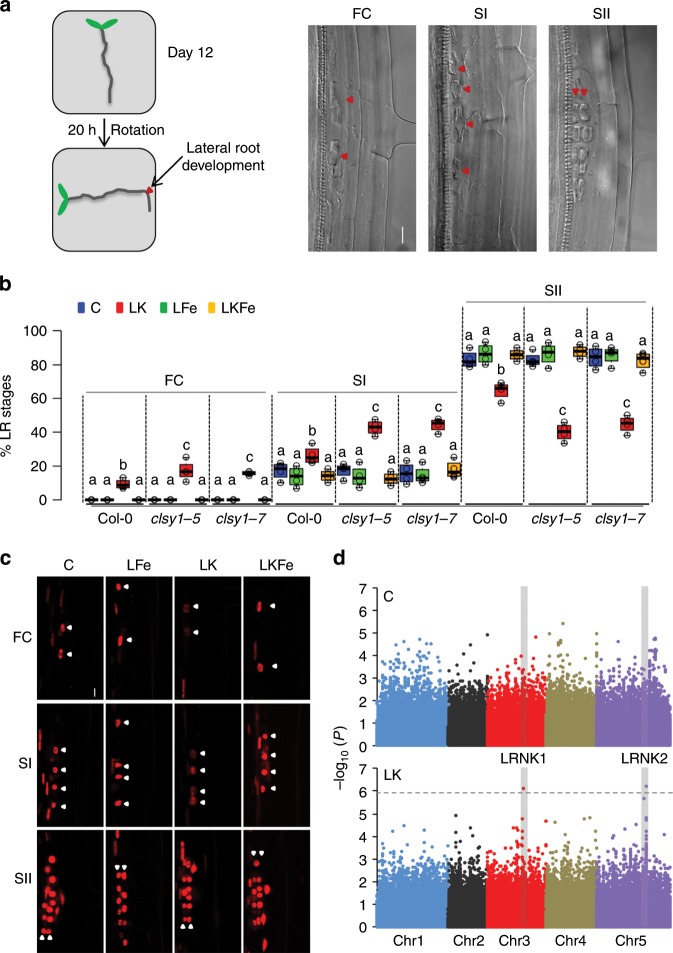
Low K inhibits the progression of lateral root primordia through early developmental stages in *Arabidopsis*. **a** Gravistimulation assay used to assess lateral root (LR) development at the outer side of an induced bend (left) and confocal microscopy images of LR founder cells (FCs), stage I (SI), and stage II (SII) primordia in *A. thaliana* Col-0 roots (right). Red triangles indicate the site where the LR develops in the assay and the cells representing different LR developmental stages. Scale bar, 10 µm. **b** LR development of Col-0 wild type and *clsy1* mutant plants in control (C, blue), low K (LK, red), low Fe (LFe, green), and low K and low Fe (LKFe, orange) conditions. The box plot gives the percentage of LRs in a particular developmental stage (FC, SI, and SII) in roots of plants from *n* = 4 independent experiments (open circles). The number of plant roots analysed per experiment in control/low Fe/low K/low KFe was 51/46/72/56 (Col-0), 55/46/85/57 (*clsy1*-*5*), 43/50/70/48 (*clsy1*-*7*). Centre lines within box plots represent sample medians. Box limits indicate the 25th and 75th percentiles; whiskers extend to 1.5 times the interquartile range from the 25th and 75th percentiles. Different letters indicate significant differences at *P* < 0.001 (one-way ANOVA) within each stage. **c** Confocal microscopy images of auxin reporter *DR5-*VENUS fluorescence (red signal) in LR cell nuclei at different stages (white triangles). **d** Manhattan plots of genome-wide association studies for LR number from 147 *Arabidopsis* accessions grown in control and low K environments. The five *Arabidopsis* chromosomes are depicted in different colours. A horizontal dashed line shows the 10% FDR threshold. The source data of **b**, **d** are provided in a Source Data file.

To gain further insight into whether the reduction of LR number in low K is a bona-fide developmental phenotype or merely a consequence of a shorter MR, we investigated the early stages of LR development after gravitational stimulation (Fig. 1a). We found that low K causes on average a 20% decrease in stage-two (SII) LR primordia and a concomitant increase in stage-one (SI) LR primordia and FCs (Fig. 1b, Col-0), indicating a post-initiation delay in the progression of LR development to SII. To assess whether auxin plays a role in the low-K phenotype, we monitored the auxin-dependent expression of *DR5*-VENUS, a synthetic fluorescent auxin reporter. Roots grown in low K show less fluorescence signal than roots grown in control conditions, specifically in LR FCs and S1 primordia (Fig. 1c, Supplementary Fig. 3) indicating either lower auxin levels or disrupted auxin signalling in these cell types. The delay of LR developmental progression in low K conditions is in good agreement with the measured ~25% decrease in the number of emerged LRs (Supplementary Fig. 1b), and shows similar Fe-dependency (Fig. 1b). We conclude that decreased LR number in low K is a macroscopic consequence of an early developmental defect and offers a convenient readout for LR development in high-throughput studies.

### *CLSY1* underpins natural variation of LR development in low K

To identify the genetic determinants of low-K induced LR inhibition, we performed a genome-wide association analysis in 147 *Arabidopsis* RegMap-panel accessions using 250 K single nucleotide polymorphisms (SNPs) (Supplementary Data 1). Two SNPs at positions 14,741,463/Chr3 (LRNK1) and 8,075,456/Chr5 (LRNK2) associate with LR number specifically under low K and moderate Fe supply (Fig. 1d, Supplementary Fig. 4). This study focusses on the LRNK1 association that lies in an intergenic region. We tested three nearby genes, *AHA8*, *EOL1* and *CLSY1* (Supplementary Fig. 5a), for a potential function in LR development in Col-0. While *aha8* and *eol1* plants exhibit a similar decrease of LR number in low K as wild-type plants (Supplementary Fig. 5b), two independent *CLSY1* knock-out lines, *clsy1-5* and *clsy1-7*, show significantly greater LR inhibition in low K (~50% decrease of LR number in *clsy1* compared to ~25% in wild type; Supplementary Fig. 5c, d). To check if *CLSY1* affects LR responses to the limitation of other major nutrients namely nitrogen (N) and phosphate (P), we assessed LR number of Col-0 wild type and *clsy1* mutant plants in low N and low P conditions. Both treatments cause a decrease in LR number which is similar in Col-0 wild type and *clsy1* mutants (Supplementary Fig. 6). In the gravistimulation assays, *clsy1* mutants show hypersensitivity of developmental defects to low K with a greater increase in FCs and SI primordia and a greater reduction in LR SII primordia compared to wild type (Fig. 1b). Thus, *CLSY1* counteracts the low-K induced inhibition of LR development in Col-0 and potentially underlies the LRNK1 association.

The LRNK1 SNP identified by GWA (A (reference) to G (non-reference) at position 14,741,463/Chr3) is located 14.4 kb downstream of the *CLSY1* coding sequence (Supplementary Fig. 5a). How does this SNP relate to *CLSY1*? A direct effect on *CLSY1* expression is unlikely since there is no clear correlation between CLSY1 mRNA abundance and LR number across the *Arabidopsis* accessions, although CLSY1 mRNA levels in the reference accessions tend to reside at the upper end of the non-reference spectrum (Supplementary Fig. 7). GWA mapping with an extended set of 156 *Arabidopsis* accessions using imputed full sequence SNPs data^12^ confirmed the position of LRNK1 and did not identify a significant SNP in closer vicinity to *CLSY1* (Supplementary Fig. 8a). To search for any SNPs within the coding region of CLSY1 that might be in linkage disequilibrium (LD) with the GWA SNP we, therefore, performed a local CLSY1-based association analysis^13^ using non-synonymous polymorphisms (Supplementary Data 2). A non-synonymous nucleotide polymorphism (A (reference) to C (non-reference)) at position 14,758,262/chr3 was found to associate with LR number variation (Supplementary Fig. 8b). This SNP is in complete LD with the LRNK1 SNP because there is not a single case of co-occurrence of the non-reference alleles (C and G, respectively) in the GWA panel of 156 *Arabidopsis* accessions or in the 874 genome sequences available from the ‘1001 Genomes’ project (Supplementary Table 1). The SNP in the CLSY1 coding sequence causes an aspartate (D) to glutamate (E) amino acid change at position 538 of CLSY1. *Arabidopsis* accessions carrying allele D, analysed alone (Supplementary Fig. 8c) or as a haplotype (Supplementary Fig. 8d), produce on average more LRs in low K than the accessions carrying allele E.

**Fig. 2:**
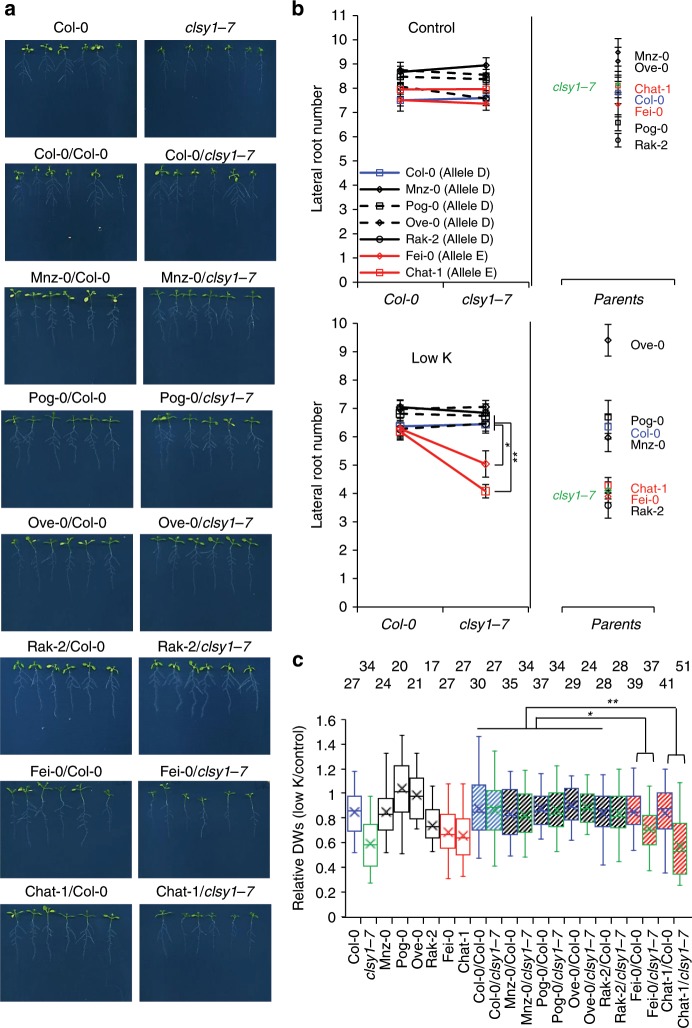
*CLSY1* allelic variation determines low-K phenotypes. Four *Arabidopsis* accessions carrying CLSY1 allele-D (Col-0, Mnz-0, Pog-0, Ove-0, Rak2; black) and two accessions carrying allele-E (Chat-1 and Fei-0; red) were crossed with Col-0 wild type (Col-0; blue) or Col-0 *clsy1-7* mutant (*clsy1-7*; green). **a** Phenotype of F1 plants grown on low K. **b** Means ± S.E of lateral root number in heterozygous F1 plants and parental genotypes from three independent experiments. Number of individual roots analysed in control/low K: *n* *=* 30/30 (Col-0 x Col-0), 32/32 (Col-0 × *clsy1*-*7*), 49/48 (Mnz-0 x Col-0), 37/45 (Mnz-0 × *clsy1*-*7*), 25/43 (Pog-0 x Col-0), 32/49 (Pog-0 × *clsy1*-*7*), 36/44 (Ove-0 x Col-0), 38/38 (Ove-0 × *clsy1*-*7*), 28/29 (Rak-2 x Col-0), 28/28 (Rak-2 × *clsy1*-*7*), 39/50 (Fei-0 x Col-0), 33/51 (Fei-0 × *clsy1*-*7*), 40/38 (Chat-1 x Col-0), 28/50 (Chat-1 × *clsy1*-*7*), 14/19 (Col-0 wild type), 18/22 (*clsy1*-*7*), 13/17 (Mnz-0), 17/9 (Pog-0), 11/17 (Ove-0), 16/16 (Rak-2), 21/23 (Fei-0), and 14/23 (Chat-1). The allele x background interaction tests were performed using two-way ANOVA, and significant differences are indicated by asterisks (*P *<* 0.05 and **P *<* 0.001). **c** Relative (low K/control) shoot dry weights (DWs) of parental and F1 plants from three independent experiments. Centre lines and crosses within the box plots represent sample medians and means. Number of roots analysed (*n*) for each genotype and condition is given above the graph. Box boundaries indicate the 25th and 75th percentiles; whiskers extend to 1.5 times the interquartile range. Statistical analysis was carried out as in (**b**). The source data of  **b**, **c** are provided in a Source Data file.

To further ascertain that *CLSY1* underlies the LRNK1 association, we performed quantitative complementation tests. Several *Arabidopsis* accessions (Supplementary Fig. 9) carrying either allele D (Col-0, Mnz-0, Pog-0, Ove-0 and Rak-2) or allele E (Chat-1 and Fei-0) were each crossed with Col-0 *clsy1*-*7* mutant or Col-0 wild type. F1 plants from these crosses were genotyped for heterozygosity (Supplementary Fig. 10) and phenotyped for LR number (Fig. 2a and Supplementary Fig. 11). The crosses reveal a significant allele-background interaction for LR number specifically in low K (Fig. 2b). F1 plants carrying a Col-0 *CLSY1* allele have similar LR numbers, whether they carry allele D or allele E on the homologous chromosome. However, allele-E/*clsy1*-*7* F1 plants (Chat-1/*clsy1-7* and Fei-0/*clsy1-7*) show significantly lower LR numbers than allele-D/*clsy1*-*7* plants (Col-0/*clsy1-7*, Mnz-0/*clsy1-7*, Pog-0/*clsy1-7*, Ove-0/*clsy1-7* and Rak-2/*clsy1-7*). The fact that *clsy1* complementation for LR number is allele-dependent rather than phenotype-dependent is apparent in the fact that the Rak-2 (D allele) complements the Col-0 *clsy1* mutant even though the parental phenotype is similar to that of Chat-1 and Fei-0 (E alleles). We propose that the D538E polymorphism, in CLSY1 underpins the observed natural variation of LR number in low K. The SNP lies in a predicted acidic domain of unknown function (Supplementary Fig. 8b), and how exactly it can alter CLSY1 structure and function remains to be investigated.

We next investigated whether genetic variation in *CLSY1* had physiological significance beyond root branching. Allele-E accessions showed a strong decrease of shoot dry weight (DW) in low K while allele-D accessions were less affected (Fig. 2c and Supplementary Fig. 12). Shoot growth inhibition by low K was more pronounced in *clsy1* than in wild-type Col-0, and was rescued by D-alleles but not by E-alleles in the F1 lines (Fig. 2c and Supplementary Fig. 12). A strong positive correlation between shoot DW and LR number, particularly under low-K (Supplementary Fig. 13) reflects the important role of root branching for plant growth. Importantly, *CLSY1* genetic variation influences LR root development and shoot growth of *Arabidopsis* in K-limited environments without affecting either phenotype under normal conditions (cryptic variation).

### *CLSY1* acts on lateral root development through *IAA27*

**Fig. 3:**
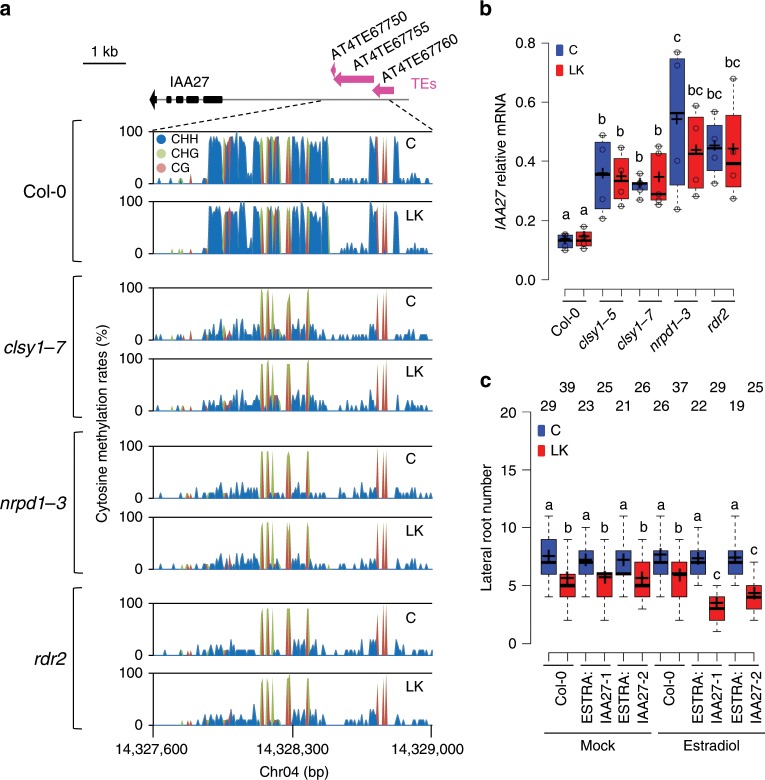
*CLSY1* acts on lateral root development by RdDM-mediated repression of IAA27. **a** Cytosine methylation rate in an upstream region of *IAA27* containing three transposable elements (TEs) in roots of Col-0 wild type, *clsy1*-*7*, *nrpd1*-*3* and *rdr2* plants grown in control (C) or low K (LK) conditions. Sequence context is shown in different colours (CHH blue, CHG green, CG red). **b** IAA27 mRNA abundance in roots of the indicated genotypes grown in control (blue) or low K (red) conditions. Box plots show IAA27 transcript levels relative to PP2A (open circles) from *n* = 5 independent experiments for *clsy1*-*7* and *n* = 4 for all other genotypes with 24 roots pooled per experiment for RNA extraction. Different letters indicate significant differences at *P* *<* 0.05 (one-way ANOVA). **c** Lateral root number of Col-0 lines expressing IAA27 under an estradiol-inducible promoter, ESTRA:IAA27-1 and ESTRA:IAA27-2. Plants were grown in four independent batches under control (blue) and low K (red) conditions in the absence (mock) or presence of 5 µM β-estradiol. Data are presented as box plots with number (*n*) of plants indicated above the boxes. Sample medians and means are represented by centre lines and crosses within the box plots. Box limits indicate the 25th and 75th percentiles; whiskers extend to 1.5 times the interquartile range from the 25th and 75th percentiles). Different letters indicate significant differences at *P* *<* 0.05 (one-way ANOVA). The source data of **b**, **c** are provided in a Source Data file.

How does *CLSY1* act on LR development? *CLSY1* is a component of a multi-protein complex required for the Pol-IV mediated production of 21-24-nt siRNA, which initiates RdDM^9^. Col-0 mutants in which other components of the same complex are inactive, *nrpd1*-*3* and *rdr2*, mimic the K-hypersensitive LR phenotype of *clsy1* mutants (Supplementary Fig. 14). We searched three independent, published DNA-methylomes of RdDM mutants^10,14,15^ for target regions related to auxin signalling. This analysis identified that a putative promoter region of a member of the IAA family, *IAA27*, which contains several TEs, consistently displayed hypomethylation in RdDM mutants. Using bisulfite-PCR on root samples, we confirmed that *clsy1*, *nrpd1*-*3* and *rdr2* mutants all had strongly reduced cytosine methylation rates in this region, particularly in a CHH context (Fig. 3a and Supplementary Fig. 15). The loss of TE methylation can influence the expression of neighbouring genes^9^. Indeed, we found that IAA27-mRNA levels are >2-fold higher in *clsy1*, *nrpd1*-*3* and *rdr2* mutant than in wild-type roots (Fig. 3b).

Neither the DNA methylation patterns nor the expression levels of *IAA27* differ between Col-0 plants grown in control or low-K conditions (Fig. 3a, b), which indicates that RdDM is not involved in signal perception per se. This was further corroborated by the lack of regulation of *IAA27* transcription in control or low K conditions by known K-transport or K-signalling component, such as *NRT1.5/NPF7*.*3*^16^*, AKT1*^17^ and *CIPK23*^18^ (Supplementary Fig. 16). *IAA27* belongs to a family of auxin co-receptors in *Arabidopsis*, several of which (*IAA3*, *IAA12*, *IAA14*, *IAA28*) negatively regulate auxin signalling targets and inhibit LR development^6^. We tested two independent Col-0 lines that over-express IAA27 under an estradiol-inducible promoter for LR phenotypes in control and low-K conditions (Supplementary Fig. 17). The IAA27 over-expression lines are hypersensitive to low K in the presence of estradiol (but not without estradiol), thus mimicking *clsy1* (as well as *nrpd1*-*3* and *rdr2)* mutants (Fig. 3c). We, therefore, propose that *CLSY1* reduces the inhibitory effect of low K on LR development by repressing *IAA27* through the RdDM pathway.

**Fig. 4:**
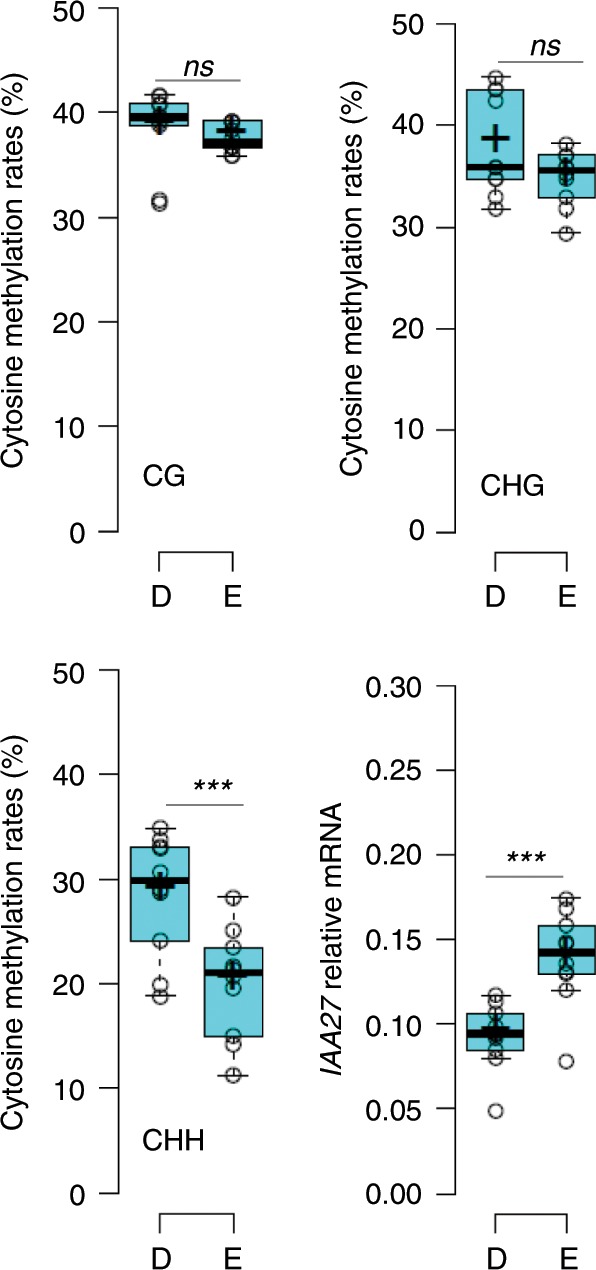
Promoter CHH methylation and expression of IAA27 differ between CLSY1 allelic variants. Average DNA methylation rates in CHH, CHG and CG context and average IAA27 mRNA abundance in *n* *=* 10 accessions carrying the CLSY1 D-allele (D) and *n* *=* 10 accessions carrying the CLSY1 E-allele (E). Cytosine methylation was determined over an upstream region of IAA27 (chr04:14327600-14329000 bp, Col-0 genome, TAIR10). IAA27 transcript levels were determined relative to PP2A using qRT-PCR on roots from three independent experiments per genotype with 18 roots per experiment pooled for RNA extraction. ***: significant difference at *P* *<* 0.005; *ns*: not significant (one-way ANOVA). Sample medians and means are shown by the centre line and crosses, respectively, box edges represent the 25th and 75th percentiles. Whiskers extend to 1.5 times the interquartile range from the 25th and 75th percentiles. The source data are provided in a Source Data file.

Does this model hold at population level? To assess the natural variation of *IAA27* promoter DNA-methylation and mRNA abundance we performed bisulfite-sequencing and RT-qPCR for IAA27 in twenty accessions, ten carrying the CLSY1 D-allele and ten carrying the CLSY1 E-allele. The average methylation rate of cytosine (specifically in CHH context) is significantly higher in the D-allele accessions than in E-allele accessions, and the average mRNA-level is significantly lower in D-allele accessions than in E-allele accessions (Fig. 4). Both entities vary between the individual accessions, as expected given the different genetic backgrounds, but they fall into distinct ranges for D and E-allele accessions (Supplementary Figs. 18 and 19). The results show that differences in *CLSY1* alter RdDM-mediated repression of *IAA27* both in mutants and in naturally occurring genotypes.

### Low K acts upstream and *CLSY1* acts downstream of auxin

Why does *IAA27* over-expression affect LR development in low K but not in control conditions? One possible explanation is that differences in *IAA27* transcription only matter when regulation at the protein level is perturbed. To monitor IAA27 protein levels in vivo we generated Col-0 lines expressing IAA27 N-terminally fused to GFP, and measured fluorescence in the roots of plants grown either in control or in low-K conditions. The fluorescence signal/background ratio from the transgenic lines was generally low but provided nevertheless statistically significant evidence for higher IAA27 protein levels in low-K roots compared to control roots, particularly in the nuclei (Supplementary Fig. 20). When we expressed GFP-IAA27 in a mutant line defective for the auxin receptor *TIR1* (*tir1-11*) we recorded a significant nuclear fluorescence signal already in control conditions, confirming impaired IAA27 degradation, and no further increase was seen when the *tir1-11* plants were grown in low K, suggesting that *TIR1* acts downstream of low K. The observations support a model in which low K prevents TIR1-dependent degradation of IAA27 protein, thereby opening an opportunity for *CLSY1* to regulate *IAA27* levels via transcriptional repression.

**Fig. 5:**
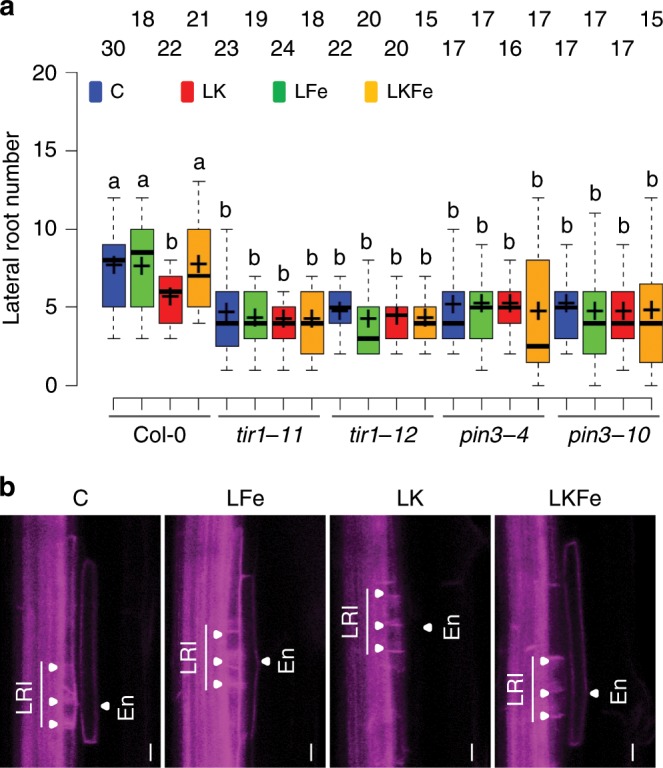
Low K acts upstream of the auxin pathway. **a** Lateral root number in mutants defective in the auxin receptor *TIR1* and the auxin efflux transporter *PIN3* grown in control (C, blue), low Fe (LFe, green), low K (LK, red), and combined low K and low Fe (LKFe, orange) conditions. Plants were grown and treated in three independent batches. The number of roots analysed (*n*) is indicated above the box plots. Sample medians and means are indicated with centre lines and crosses, respectively, and box edges represent the 25th and 75th percentiles. Whiskers extend to 1.5 times the interquartile range from the 25th and 75th percentiles. Different letters indicate significant differences at *P* *<* 0.05 (one-way ANOVA). **b** Confocal microscopy images showing expression of a prPIN3:PIN3-GFP fusion protein in the roots of Col-0 plants grown in the indicated nutrient conditions. White triangles point to LR initials (LRI) and overlaying endodermal (En) cells. The source data of **a** are provided in a Source Data file.

At which point does low K interfere with the auxin pathway? Mutants defective for the IAA27-interacting transcription factor ARF7 (*arf7*)^19,20^, the auxin receptor *TIR1* (*tir1-11*, *tir1-12*) and the auxin transporter, *PIN3*, (*pin3-4*, *pin3-10*) all have a constitutive LR developmental phenotype, forming less LRs than wild type in control, and not responding to K availability (Fig. 5a, Supplementary Fig. 21). Thus, low K acts upstream of auxin transport and perception. PIN3 enables auxin reflux into the developing LRs from the overlaying endodermis cells^21^. Confocal microscopy of a Col-0 line expressing pPIN3::PIN3-GFP revealed that low K eliminates PIN3-GFP expression specifically in the endodermis cells that overlie the LR initials (Fig. 5b and Supplementary Fig. 22). Again, this effect was not seen when Fe was lowered together with K. Less auxin accumulation in LR primordial cells under low K is also consistent with the earlier experiments showing less *DR5*-VENUS fluorescence in FCs and SI-primordia (Fig. 1c).

**Fig. 6:**
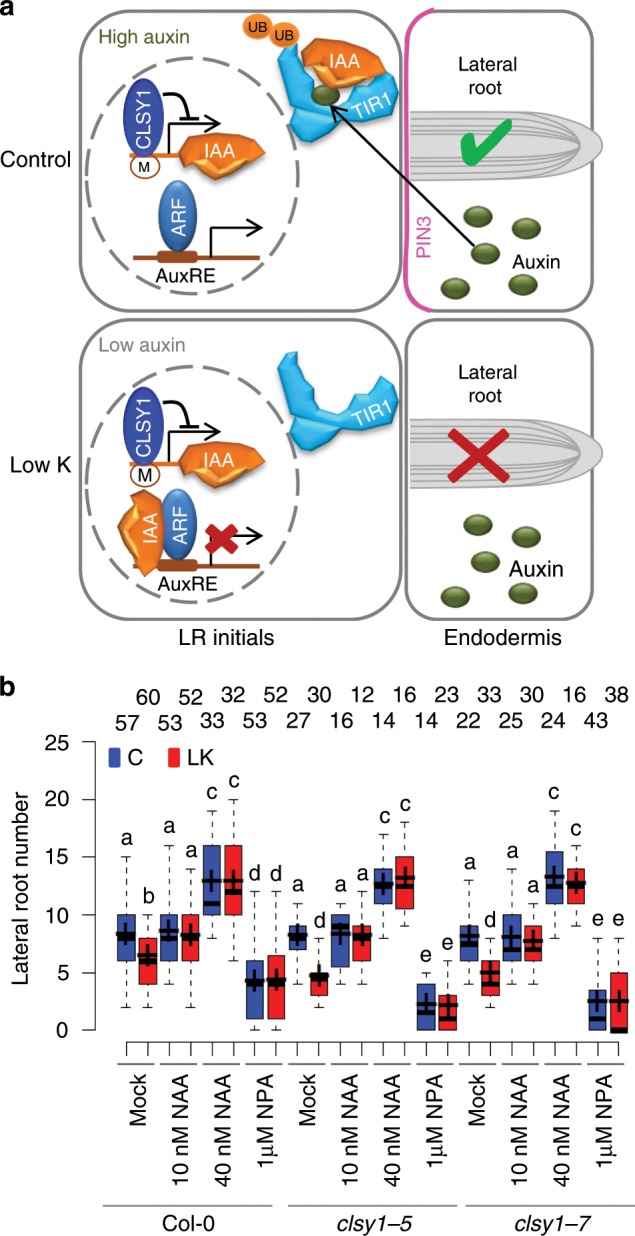
*CLSY1* maintains lateral root development when the auxin pathway is perturbed exogenously. **a** Schematic summary of the proposed role of *CLSY1* at cellular level. In normal conditions, IAA27 is bound by the auxin-TIR1 complex, and targeted for degradation, which releases IAA-inhibition of ARFs, and promotes LR development. When this pathway is perturbed, for example by reduced auxin reflux from PIN3 expressing endodermal cells under low K, LR development depends on transcriptional repression of IAA27, which is mediated by *CLSY1*. **b** Number of lateral roots in Col-0 and *clsy1* plants grown under control or low K conditions, in the absence or presence of indicated concentrations of auxin analogue NAA or auxin inhibitor NPA. The number of roots analysed (*n*) is indicated above the box plots. The centre lines of the boxplots correspond to medians and the crosses to the means. The boundaries of the box are the first and third quartiles and whiskers extend to 1.5-fold the interquartile range. Significant differences at *P* *<* 0.05 (one-way ANOVA) are indicated by different letters. The source data of **b** are provided in a Source Data file.

Based on the mutant phenotypes and the microscopy analyses we propose that low K, in an Fe-dependent manner, interferes with cell-type specific PIN3 expression and reduces auxin reflux to LR initials, thereby perturbing auxin-dependent proteolysis of IAA27 and downstream signalling (Fig. 6a). We further tested this model with pharmacological assays. As would be expected, exogenous application of synthetic auxin (1-naphthaleneacetic acid, NAA) counteracts the effect of low K and of the *clsy1* mutation on LR growth, while inhibition of auxin transport with a phytotropin (naphthylphthalamidic acid, NPA) makes LR development sensitive to *CLSY1*-function, even in K-sufficient conditions (Fig. 6b and Supplementary Fig. 23).

**Fig. 7:**
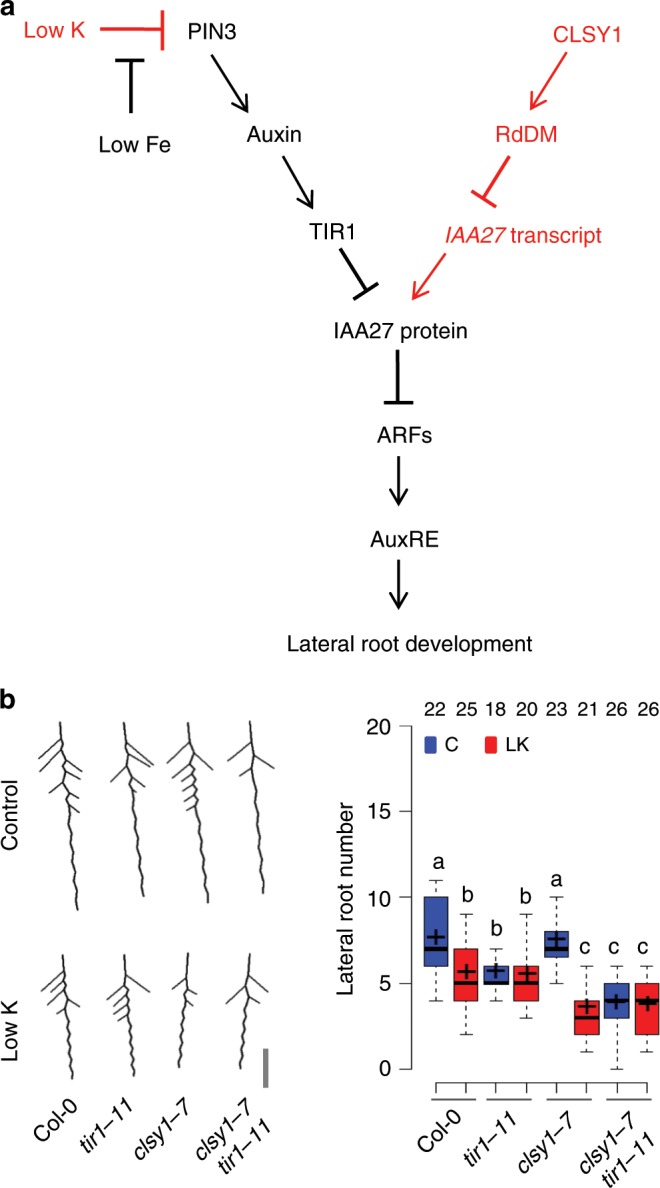
*CLSY1* maintains lateral root development when the auxin pathway is genetically disrupted. **a** Schematic summary of the proposed interaction between the canonical auxin pathway (black) and the *CLSY1*-RdDM mediated backup pathway (red). Existence of the latter only becomes apparent when the auxin pathway interrupted by environmental or genetic perturbation. **b** Average root phenotypes and lateral root number of Col-0 wild type, *clsy1*, *tir1* and *clsy1tir1* double mutants in control (C, blue) and low K (LK, red) conditions. The depicted average root architectures (scale bar 1 cm) were generated with EZ-Root-Vis software from individual plants grown in three independent experiments. The number of plants analysed (*n*) is indicated above the box plots. The centre lines are the medians of the data and crosses are the means; box edges are the first and third quartiles; the whiskers extend to 1.5 time the interquartile range. Different letters denote significant differences at *P* *<* 0.05 (one-way ANOVA). The source data of **b** are provided in a Source Data file.

The combined findings provide strong evidence that *CLSY1*-mediated RdDM and transcriptional repression of *IAA27* constitute an independent backup pathway that regulates LR development when the canonical auxin pathway is perturbed (Fig. 7a). The model predicts that *CLSY1* would also protect LR development against genetic disruption of the auxin pathway upstream of IAA27 degradation, even when K supply is sufficient. To test this hypothesis, we generated a *clsy1*-*7 tir1*-*11* double mutant. The *TIR1* mutation inhibits LR root development more strongly in the *clsy1* than in the Col-0 wild type background, in both control and low K conditions, and has no additive effect to *clsy1* in low K (Fig. 7b). The results confirm that *CLSY1* maintains LR development process when the auxin pathway fails, irrespective of whether the perturbation is caused by an environmental or genetic challenge.

## Discussion

K is an essential mineral nutrient required for many fundamental processes of life including electric, osmotic and metabolic functions^22^. It is highly soluble and therefore leaches from soils with low colloid or clay content or from clay-rich soils with poor structure^23^. In agriculture, particularly in developing countries, K management is often neglected in favour of nitrogen and phosphorus, but once soils have become K-depleted soil health cannot easily be restored. K deficiency, therefore, limits natural vegetation and crop production in many parts of the world^24^. Even in soils that have overall sufficient K, high K demand during certain developmental stages (e.g. early growth or seed filling) can lead to local K depletion in densely-rooted areas^23^. Whether roots avoid or outgrow depletion zones and how this affects plant performance is an important question for agriculture and for ecology, calling for a more detailed understanding of root developmental responses to low K.

We have shown here that low K in the root environment inhibits early LR development by causing a delay in the progression from FC towards stage-II LR primordia. Genetic and microscopic evidence revealed that low-K disrupts the auxin-dependent initiation of LR development upstream of auxin perception by TIR1 and interferes with the expression of PIN3 transporter proteins and with auxin accumulation in the LR primordia (Fig. 6a). The exact mode of action remains to be elucidated. Other K-deficiency responses involve the K-channel AKT1 as a ‘transporter-receptor’ in conjunction with a calcium signalling module consisting of calcineurin-B like (CBLs) calcium-binding proteins and CBL-interacting protein kinases (CIPKs). Mutants in these components are generally smaller in low K than wild-type plants and show some changes in root architecture. Whether AKT1/CBL/CIPK signalling is also required for the low-K response of early LR development and for PIN3 expression remains to be explored. In analogy with previously reported Fe-dependence of MR inhibition by low phosphate the observed Fe-dependence of LR-inhibition by low K suggests that the molecular events upstream of PIN3 expression include Fe-dependent redox processes. These could either directly impact on protein translation and quality control in the ER or be part of a signalling pathway involving reactive oxygen species (ROS). An increase of ROS in K-deprived roots has been reported^25^. Whether and how redox potentials and/or ROS signalling contribute to the early LR developmental phenotype in low K remains to be investigated.

In this study we focussed on the question what determines the extent of LR suppression in low K. The vast majority of accessions analysed here have lower LR number in low K than in sufficient K, but the severity of the inhibition varies. Robustness has been defined as invariant phenotype under environmental perturbation^1^ and requires mechanisms to buffer or over-ride sensitivity. We found that the strategy used by plants to maintain LR development in low K is similar to how engineers introduce robustness into control systems. *CLSY1*-mediated RdDM does not alter the K-sensitivity of the auxin pathway *per se* but generates a bypass circuit that directly controls an important switch at the end of the pathway (*IAA27*). When the auxin pathway is off (low K or *tir1*) and the backup is on (D-allele genotypes), *CLSY1*-mediated transcriptional repression maintains low IAA27 levels, thereby releasing inhibition of ARFs and maintaining LR development (Fig. 6a). This model currently offers the best explanation for the combined evidence from our experiments, but proof of quantitative differences in IAA protein levels in vivo is technically challenging. The GFP-IAA27 expressing lines generated here show increased IAA27 protein levels in Col-0 wild type in low K compared to control, and constitutively high IAA27 protein levels in Col-0 *tir1* mutants, which is in accordance with the model. However, the signal/background ratio is still low preventing quantitative comparison between different lines. Stronger and ratio-metric reporters are needed in the future, and they would have to be expressed in the background of different accessions and mutants to fully prove our model.

While *CLSY1* is an important determinant of LR development when the auxin pathways is interrupted, it is not the only one. Accessions with a CLSY1 D-allele showed on average higher CHH methylation in a TE-containing upstream region of *IAA27*, lower IAA27 mRNA levels and more LRs in low K than accessions with an E allele. Crossing D-alleles from different accessions into the Col-0 *clsy1* mutant rescued the K-hypersensitive phenotype whereas crossing with E-alleles did not, confirming that D is the functional allele. Nevertheless, all three readouts (DNA methylation and IAA27 mRNA, and LR number in low K) still varied considerably among accessions with the same CLSY1 allele indicating that each of them is also regulated by other factors, which differ between accessions. GWA statistics can extract individual pathways even if they are buried in complex networks and allowed us to separate the mechanism that links *CLSY1* with LR number from a larger network of interactions. To further unravel this network it would be informative to characterise those accessions in which individual links between DNA-methylation, transcript levels and LR number are broken and to identify the genes and pathways that are responsible.

An obvious question is why natural genetic variation has targeted *CLSY1* rather than *IAA27* itself. Possible answers range from positional preferences to functional advantages. DNA-methylome sequencing indicates several hundred potential gene targets of RdDM in Col-0 and the target subsets differ for different *CLSY* isoforms^10^. IAA27 was the only obvious candidate relating to LR development that is consistently shown to be a target of RdDM in Col-0 accession across independent studies^10,14,15^ (Fig. 3a), but it would be interesting to explore whether the CLSY1 D/E polymorphism leads to other cryptic phenotypes and whether there is a functional bias among CLSY1 targets. One possibility is that RdDM-mediated transcriptional silencing more generally provides a backup system for processes that involve protein degradation of negative regulators (double-negative regulation), which is a typical feature of hormonal regulation in plants. It is worth noting that in most cases the transcriptional repression of a negative regulator will only affect protein levels when the canonical protein degradation pathway is perturbed, and its effect is, therefore, conditional by nature. By contrast, changing transcription rate of a positive regulator is more likely to have constitutive effects under normal conditions. The advantage of employing an RdDM-mediated backup circuit to achieve robustness is, therefore, likely to be restricted to double-negative regulation and could have contributed to its evolution.

Does *CLSY1*-mediated robustness of LR development give plants an advantage, and under which conditions? Our analysis of *Arabidopsis* seedlings grown on moderately low K for 2 weeks revealed higher shoot weights of plants carrying the CLSY1 D-allele than of plants carrying the E-allele. We also found a positive correlation between LR number and shoot weight across tested genotypes, which is not surprising given the important role of LRs in uptake of water and nutrients. Therefore, at least in the case of moderate or short-term K deficiency continuing LR development seems to be an advantage. Manipulating transcript levels of *IAA27* could offer an opportunity to increase the robustness of lateral root development and improve plant performance in low-K environments that are characterised by patchy or transient K-deficiency. Seen in this light it makes sense that *CLSY1* itself is not regulated by environmental cues as it would be difficult for the plant to sense the wider geographical or temporal context of K-deficiency. By contrast, this context could have contributed to evolution of *CLSY1* and its natural variation. Unfortunately, insufficient information is available on the habitats from which the accessions used in this study were collected. New accessions with detailed metadata for the specific soil properties and temporal patterns of K supply should be collected in the future to determine the exact scenarios under which continued LR development under K-deficiency is advantageous.

In summary, our study reveals that cryptic natural variation in the RdDM machinery defines the balance between robustness and plasticity in the face of environmental and genetic perturbation. Gene repression through RdDM provides a backup system for maintaining lateral root development when hormone-mediated protein degradation is impaired, thus enabling plants to adopt a root system architecture that favours growth in a challenging environment.

## Methods

### Plant materials and growth conditions

*Arabidopsis thaliana* accessions used in the study comprised 139 accessions shared between the RegMap^26^ and 1001 genome^12^ panels, and 8 accessions specific to RegMap and 17 accessions specific to 1001 genome panel. The seeds were obtained from Nottingham *Arabidopsis* Stock Centre (NASC). The homozygous T-DNA insertion mutant lines for several genes, namely *CLSY1* (*clsy1*-*7* (SALK_018319) and *clsy1*-*5* (SAIL_1229_H10), *TIR1*(*tir1*-*11* (SALK_090445C), *tir1*-*12* (SALK_151603C)), *PIN3* (*pin3*-*4* (N9363), *pin3*-*10* (SALK_113246C)), *NRPD1a* (*nrpd1*-*3* (SALK_128428)), *RDR2* (Sail_1277_H08), *nrt1*.*5*-*2* (SALK_005099), and *cipk23*-*1* (SALK_032341) were also obtained from NASC. Previously described *akt1*^4^ and *arf7*-*1*^27^ mutant were also used during this study. The *clys1 tir1* double mutant was generated by crossing *clsy1*-*7* and *tir1*-*11* single mutant plants. Transgenic lines (ESTRA:IAA27-1 (N2102233) and ESTRA:IAA27-2 (N2102234)) expressing IAA27 transcription factor under the control of beta(β)-estradiol inducible promoter were obtained from the TRANSPLANTA collection^28^.

Surface sterilization of seeds was performed using 1 min wash with absolute ethanol followed by 5 min wash with a solution containing 2.8% sodium hypochlorite and 0.1% Tween^**®**^-20. The seeds were then washed five times using sterile distilled water. The seeds were stratified at 4 °C for 5–7 days and then sown on vertical plates with minimal media^4^ containing 1% agar (Formedium), 0.5% sucrose and pH 5.6 (0.2 M MES/Tris). Control media contained 0.5 mM NaH_2_PO_4_, 2 mM KNO_3_, and 0.25 mM MgSO_4_, 0.25 mM MgCl_2_, 2 mM CaCl_2_, and 42.5 µM Fe(III) Na-EDTA, as well as 1.8 µM MnSO_4_, 45 µM H_3_BO_3_, 0.38 µM ZnSO_4_, 0.015 µM (NH_4_)6Mo7O_24_, 0.16 µM CuSO_4_, and 0.01 µM CoCl_2_. For low K and/or low Fe experiments, concentrations of K and Fe were lowered to 10 and 4.25 µM, respectively. The osmotic potential of these media was adjusted with NaCl. Low N and low P media contained 50 and 20 µM of N and P, respectively. Plants were grown for 12 days in a growth chamber at 60% relative humidity and 22 °C, with cycles of 9 h light (120 µE m^−2^ s^−1^). The expression of *IAA27* in the IAA27 over-expressing lines was induced by growing plants for 12 days on media supplemented with 5 µM β-estradiol (Sigma Aldrich, cat#E2758). For treatments with auxin efflux inhibitor (N-1-naphthylphthalamidic acid (NPA) (1 µM) (Santa Cruz Biotechnology, cat#132-66-1)) or synthetic auxin (1-Naphthaleneacetic acid (NAA) (10 nM and 40 nM) (Sigma Aldrich, cat#317918)), seeds were germinated and grown for 12 days on media supplemented with these chemicals.

### Root phenotyping

Images of plants on plates were acquired using a flatbed scanner at 200 dpi^29^. Lateral root number, main root length, and total root size of individual plants were quantified from the images using a semi-automated pipeline in the EZ-Root software^30^. Statistical analysis and visualization of average root system architectures was carried out using Root-Vis software^30^.

Gravistimulation assays were performed to visualize the dynamics of LR development responses to K and Fe availability. This method helps to synchronize the location and timing of LR primordia initiation^27^ by inducing an auxin maxima at the outer site of the bend^31^. Plates with 12-d-old plants were rotated by 90°, and the plants were allowed to grow for another 20 h. Plant roots were then mounted in the clearing solution (1 M chloral hydrate and 33% glycerol) and observed after 10 min with Zeiss LSM510 microscope using a white field camera.

### Genetic mapping

For genome-wide association (GWA) analysis, LR number data were obtained from two sets of *Arabidopsis* accessions: (i) 147 accessions belonging to RegMap panel^26^; and (ii) 156 accessions from 1001 genome panel^12^, each with an average of five replicates (Supplementary Data 2). 139 *Arabidopsis* accessions were shared between the two panels. GWA mapping was performed on mean LR number data using the GWAPP web interface (https://gwas.gmi.oeaw.ac.at/) and the accelerated mixed-model (AMM) method^32^. Genotype data from 250k single nucleotide polymorphism (SNP) chip^26^ and imputed full sequences^12^ were respectively used for mapping in the RegMap and 1001 genome panels, and only SNPs with minimum allele frequency (MAF) > 0.05 were considered. In both GWA analyses, the LR number-associated SNP under low K environment identified at position 14,741,463/Chr3 (LRNK1) was located 14.4 kb downstream of the CLSY1 coding region, whereas, closer SNPs did not show any significant association. To clarify, we performed CLSY1-based local association analysis. The coding DNA sequences of CLSY1 from 156 accessions were retrieved from Salk *Arabidopsis* 1001 Genomes database (http://signal.salk.edu/atg1001/index.php). Association analysis for LR number variation was performed using 13 non-synonymous SNPs (MAF > 0.05) with Tassel 5 using generalized linear model^33^. CLSY1 haplotypes were defined based on these polymorphisms.

### Quantitative complementation

To verify that *CLSY1* underlies LR number association under low K environment (LRNK1), we performed quantitative complementation tests^34^. These tests allow the effects of natural alleles to be determined in a heterozygous state with a null allele. Moreover, the phenotypes of transgenic *Arabidopsis* plants tend to be highly variable due to experimental uncertainties, such as the copy number, transgene expression levels, and the insertion sites. *Arabidopsis* accessions Col-0, Mnz-0, Pog-0, Ove-0, and Rak-2 corresponding to CLSY1 allele D, and the accessions Chat-1 and Fei-0, which carry the allele E, were each crossed with either *clsy1*-*7* mutant or its wild type (Col-0) background. F1 plants from these crosses were genotyped using the indel markers to ensure that they had the expected allelic combinations. Primer sequences for indel markers are provided in Supplementary Table 2. F1 plants along with parental accessions were phenotyped for LR number and shoot dry weight (DWs). Allele-to-background interactions were assessed using Two-way analysis of variance (ANOVA).

### Quantitative gene expression analysis

*CLSY1* and IAA27 mRNA abundance was characterized in natural *Arabidopsis* accessions or in T-DNA lines using quantitative real-time (qRT-PCR). Total RNA was extracted from pools of 18–24 plant roots (see figure legends) using a SV Total RNA Isolation System (Promega, cat#Z3101). One µg of total RNA was used for first strand cDNA synthesis using M-MLV Reverse Transcriptase, RNase H Minus, Point Mutant (Promega, cat#M3681) and Oligo(dT)15 Primer (Promega, cat#C1101) in a final volume of 25 μL, according to the manufacturer’s instructions. 25 ng of first strand cDNA was used as template for qRT-PCR. qRT-PCRs were performed in StepOnePlus RT-PCR system (Applied Biosystems). cDNA amplification was monitored using SYBR Green (Clonetech, Cat#RR820Q) at an annealing temperature of 55 °C. *PP2A* (AT1G13320) was used as an internal control. The primer sequences used are listed in Supplementary Table 2. The PCR efficiency (E) for each primer pair was determined after the analysis of 1∶4, 1∶16, 1:64 and 1:256 dilutions of first strand cDNA by using the equation E = (10^−1/s^), where s is the slope of the linear regression of the threshold cycle (Ct) values. Relative transcript levels of *IAA27* compared to the *PP2A* were determined using the equation *RTL* = [(E)^−Ct^]_*IAA27*_ /[(E)^−Ct^]_*PP2A*_^35,36^.

### Bisulfite sequencing

Genomic DNA was extracted from roots of Col-0, *clsy1***-***7*, *nrpd1*-*3*, and *rdr2* plants using DNeasy Plant Mini kit (Qiagen, cat#69104), following the manufacturer’s instructions. Three independent experiments were performed each comprising pooled samples of 24 plant roots. 0.5 µg of genomic DNA was treated with bisulfite using the EpiTect Bisulfite kit (Qiagen, cat#59104). The primers listed in Supplementary Table 2 were used to amplify the IAA27 promoter region corresponding to TEs (AT4TE67750, AT4TE67755, and AT4TE67760). The fragments were then cloned in pDONR207 and in total 10 (at least 3 from each experiment) clones for each genotype were sequenced individually. The sequences were aligned using MEGA version 7.0.26^37^, and the methylation rates of individual cytosines were calculated through bisulfite conversion rate in percentage. DNA methylation rates of IAA27 promoter in natural accessions of *Arabidopsis* were monitored in the same manner.

### Confocal microscopy

*Arabidopsis* Col-0 plants expressing 3XVENUS-N7 under the control of auxin-responsive promoter pDR5^38^ were used to study the effects of K and Fe availability on transcriptional responses to auxin. The pDR5::3XVENUS-N7 fluorescence was visualized using confocal laser-scanning microscopy (Zeiss LSM510). Plant roots were illuminated with a 488 nm laser and light was collected using LP560 emission filter. For quantitative comparison of pDR5 activity, the laser power, pinhole and gain settings of the confocal microscope were identical among different treatments. The fluorescence signal intensity in cell nuclei was quantified with ImageJ. The regulation of PIN3 spatial expression pattern by K and Fe availability was studied using pPIN3::PIN3-GFP fusion protein^39^. Confocal microscopy was performed using a Leica SP8 microscope. Plant roots were imaged with a 488 nm argon laser line for excitation of GFP fluorescence. Emissions were detected between 505 and 530 nm.

### Statistical analysis

Unless stated otherwise, the statistical significance of differences between two means was assessed using one-way ANOVA. Multiple comparisons were analysed using least significant differences test. Statistical analyses were performed, and results plotted using *SigmaPlot 11.0*. Centre lines and crosses within box plots, respectively, represent sample medians and means. Box limits indicate the 25th and 75th percentiles; whiskers extend 1.5 times the interquartile range from the 25th and 75th percentiles. Treatments were generally repeated at least three times with the number of independent biological replicates (number of individual roots *n*) indicated in the figure legends.

### Accession numbers

Sequence data from this article can be found in the *Arabidopsis* Genome Initiative or GenBank/EMBL databases under the following accession numbers: AT3G42670 (*CLSY1*), AT4G29080 (*IAA27*), AT3G62980 (*TIR1*), AT1G70940 (*PIN3*), AT5G20730 (*ARF7*), AT1G63020 (*NRPD1a*), AT4G11130 (*RDR2*), AT2G26650 (*AKT1*), AT1G30270 (*CIPK23*), and AT1G32450 (*NRT1*.*5*/*NPF7*.*3*).

### Reporting summary

Further information on research design is available in the Nature Research Reporting Summary linked to this article.

## Supplementary information

Supplementary Information

Peer Review File

Reporting Summary

Description of Additional Supplementary Files

Supplementary Dataset 1

Supplementary Dataset 2

Source Data File

## Data Availability

Data supporting the findings of this work are available within the paper and its Supplementary Information files. A reporting summary for this article is available as a Supplementary Information file. Datasets generated and analysed during the current study are available from corresponding authors upon request. The source data underlying Figs. 1b, 1d, 2b, 2c, 3b, 3c, 4, 5a, 6b, and 7b as well as Supplementary Figs. 1b–d, 2b, 3, 4, 5b, 5c, 6a, 7, 12–19, 20b, 21, and 22 are provided as a Source Data file.
