# Supplementary material for: Cryptic variation in RNA-directed DNA-methylation controls lateral root development when auxin signalling is perturbed
Source: Nat Commun. 2020 Jan 10;11:218. doi: 10.1038/s41467-019-13927-3 (PMC6954204; doi:10.1038/s41467-019-13927-3)
Supplement: Supplementary file 1 — Supplementary Information [file 41467_2019_13927_MOESM1_ESM.pdf]

Cryptic variation in RNA-directed DNA-methylation controls lateral root development when auxin signaling is perturbed

Shahzad *et al.*

Supplementary Table 1. Quantitative analysis of linkage disequilibrium in *Arabidopsis* accessions.

Co-occurrence of SNPs in the 156 *Arabidopsis* accessions used for GWAS in this study:

GWA SNP	CLSY1 associated SNP	*N*
A	A	120
A	C	25
G	A	11
G	C	0

Co-occurrence of SNPs in the 874 *Arabidopsis* accessions available from the 1001 Genomes project:

GWA SNP	CLSY1 associated SNP	*N*
A	A	688
A	C	152
G	A	33
G	C	0

Note: Co-occurrence of GWA SNP and local CLSY1 SNP. *N* indicates the number of accessions carrying a specific combination of the two polymorphisms.

Supplementary Table 2. **List of primer sequences used in the study**.

Primer name	Primer sequence	Primer use for
SALK_018319-LP	AAAAGCTCCTGAGGGTTGAAG	Genotyping T-DNA line
SALK_018319-RP	TTTCTCGCGAGCTACTTGAAG	
SAIL_1229_H10-LP	CTCTGAAGTCTCGCCATCATC	Genotyping T-DNA line
SAIL_1229_H10-RP	ATTGTGACGGATGAAGATTGC	
SALK_090445C-LP	CACGTGTCATCATCAGAATCG	Genotyping T-DNA line
SALK_090445C-RP	ATTTCCCACCTCAGGAGATTC	
SALK_151603C-LP	TTTATGAATCCAAGTGGTGGG	Genotyping T-DNA line
SALK_151603C-RP	ATACACGTAACCTCCCCATCC	
SALK_113246C-LP	TGCCACCTTCAATTCAAAAAC	Genotyping T-DNA line
SALK_113246C-RP	TGATTTTCTTGAGACCGATGC	
LBb1.3	ATTTTGCCGATTTCGGAAC	T-DNA
SailLB	GCCTTTTCAGAAATGGATAAATAGCCTTGCTTCC	T-DNA
CLSY1qRTF1	CGATTACAGCCTGTGCAGAA	CLSY1 qRT PCR
CLSY1qRTR1	CTGGAGACCCATTCTTTCCA	
PP2AqrtF	TAACGTGGCCAAAATGATGC	PP2A qRT PCR
PP2AqrtR	GTTCTCCACAACCGCTTGGT	
IAA27qRTF2	GGTGTGGCAGAGATGGGTTA	IAA27 qRT PCR
IAA27qRTR2	TCTCAGCTTCTTGCAGGAGC	
ColFeiF	CTACTATTGAACATGAGGGGAAC	IND3.14752 marker
ColFeiR	TTTATTGATGCCATAGTGAAGCC	
ColchtovepogF	TCTCGATCCTTAAGTACTCCAC	IND3.14759 marker
ColchtovepogR	AGCATTACTTTGAGTTTAACCATC	
ColRakF	ATTTTCTGAACCAAAGTGTGCCAG	IND3.15132 marker
ColRakR	GTCTTGATCGTATTAACGCAG	
ColMnzF	AAGAGAATCAAACCGAGGTAATCC	IND3.14766 marker
ColMnzR	AGACTTCGCCGGAGAACATGCAAC	
IAA27cNterGFP_F	GGGGACAAGTTTGTACAAAAAAGCAGGCTTAATGTC TGTATCTGTAGCA	prUBQ:GFP-IAA27 cloning
IAA27NterGFP_R	GGGGACCACTTTGTACAAGAAAGCTGGGTACTAGTT CCTGCTTCTGCA	
IAA27bpcrF1	GGGGACAAGTTTGTACAAAAAAGCAGGCTTAATTTA ACATCCATACAAGAACCTCAT	Bisulfite PCR
IAA27bpcrR1	GGGACCACTTTGTACAAGAAAGCTGGGTATAATAAG ATGGAGTGTATA	
IAA27bpcrF2	GGGGACAAGTTTGTACAAAAAAGCAGGCTTAATTAA AACGCATAGTTTGCTAAT	Bisulfite PCR
IAA27bpcrR2	GGGACCACTTTGTACAAGAAAGCTGGGTAAATTAGT ATATGAAGTCGTTAAATA	

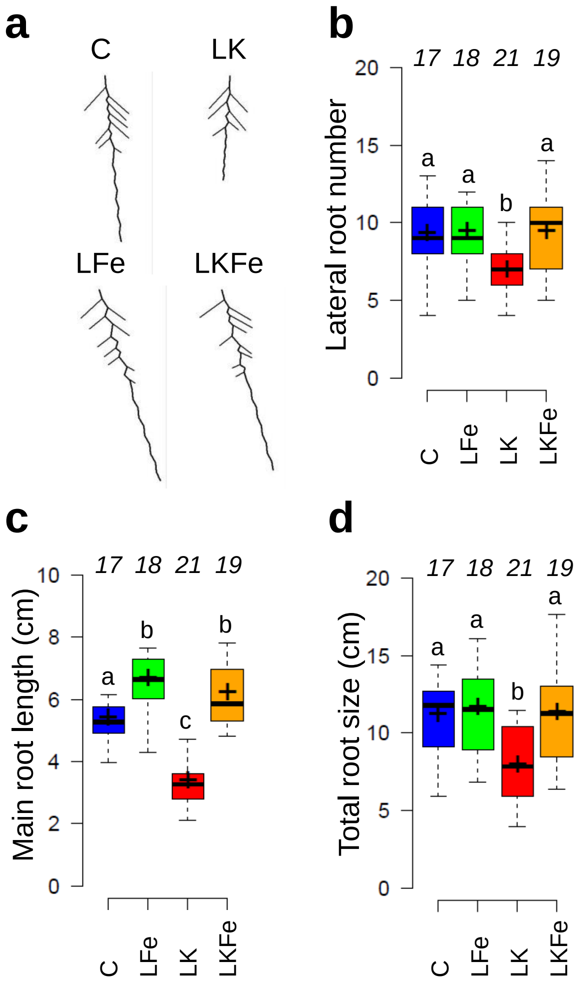

Supplementary Figure 1. **Root responses to K and Fe availability in *A. thaliana* Col-0. a**. Visual reconstructions of average root system architecture generated using the EZ-Root-VIS software[1]. **b-d.** Box plot representation of quantitative data for lateral root number (**b**), main root length (**c**), and total root size (**d**). Data are for the indicated number of plants (*n*) grown in four independent experiments in control (C, blue), low Fe (LFe, green), low K (LK, red), and low K and low Fe (LKFe, orange) conditions. Centre lines and crosses in the box plots represent sample medians and means, respectively. Box limits indicate the 25th and 75th percentiles; whiskers extend 1.5 times the interquartile range from the 25th and 75th percentiles. Letters denote significant differences at $P < 0.005$ (one-way ANOVA). The source data of Supplementary Fig. 1b-d are provided in a Source Data file.

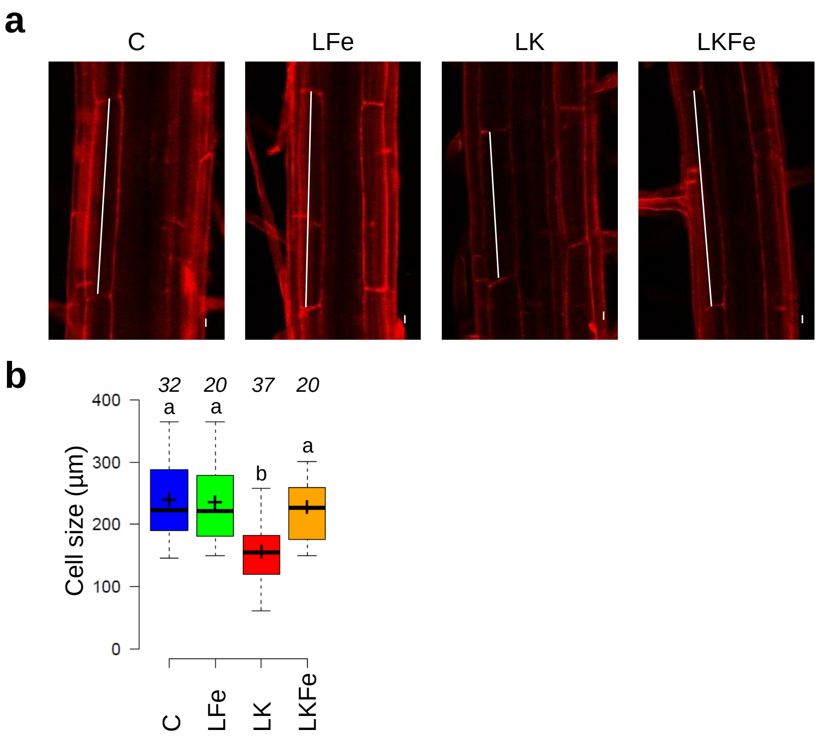

Supplementary Figure 2. **Effects of K and/or Fe availability on cell length in the mature zone of *Arabidopsis* Col-0 roots.** Propidium iodide staining was performed to visualize the cells using confocal laser scanning microscope on 12-d-old roots. **a.** Representative root images are shown. Scale bars at the right bottom of the images are 10 µm. **b.** Quantitative data for cell length of plants grown in control (C, blue), low Fe (LFe, green), low K (LK, red), and low K and low Fe (LKFe, orange) conditions are presented in box plots. The number of phenotyped cells (*n*) is indicated above the boxes. Centre lines and crosses in the boxes represent sample medians and means, respectively. Box limits indicate the 25th and 75th percentiles; whiskers extend 1.5 times the interquartile range from the 25th and 75th percentiles. Different letters indicate significant differences at $P < 0.01$ (one-way ANOVA). The source data of Supplementary Fig. 2b are provided in a Source Data file.

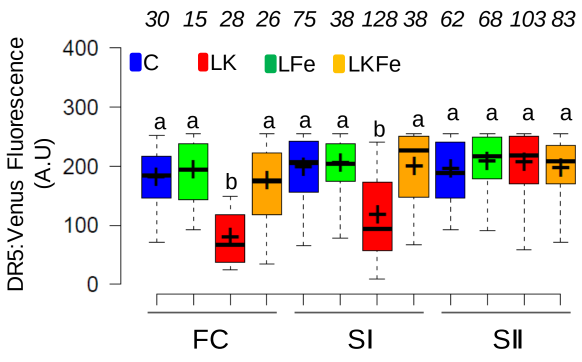

Supplementary Figure 3. **Effects of K and/or Fe availability on the activity of the auxin-responsive *DR5* promoter.** Confocal laser scanning microscope was used to visualize *DR5*-VENUS fluorescence in founder cell (FC), stage I (SI), and stage II (SII) primordia of lateral root (LR) development on 12-d-old roots of *Arabidopsis* Col-0 accession. Data for plants grown in control (C, blue), low Fe (LFe, green), low K (LK, red), and low K and low Fe (LKFe, orange) conditions are shown. The signal intensity in the nucleus (in arbitrary units, A.U.) of indicated number (n) of cells from four independent experiments at various stages of LR development was quantified using ImageJ and the data are shown as box plots. Centre lines and crosses in the boxes represent sample medians and means, respectively. Box limits indicate the 25th and 75th percentiles; whiskers extend 1.5 times the interquartile range from the 25th and 75th percentiles. Datasets for each LR developmental stage were treated separately in the statistical analysis (different letters indicate significant differences at $P < 0.001$ (one-way ANOVA)). The source data are provided in a Source Data file.

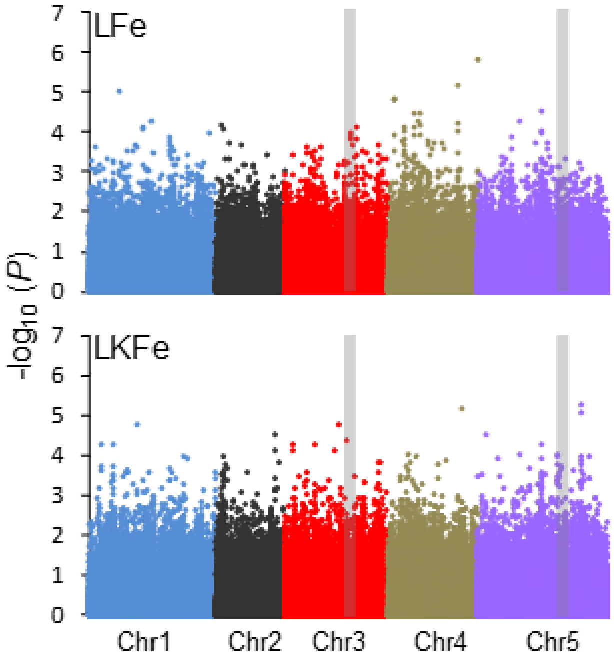

Supplementary Figure 4. Manhattan plots of genome-wide association studies for LR number from 147 *Arabidopsis* accessions grown in low Fe and low KFe conditions. LFe: low Fe; LKFe: low KFe. Vertical grey bars correspond to the position of LRNK1 and LRNK2 associations identified specifically in low K and moderate Fe conditions (see Fig. 1d). The five *Arabidopsis* chromosomes are shown in different colors. The source data are provided in a Source Data file.

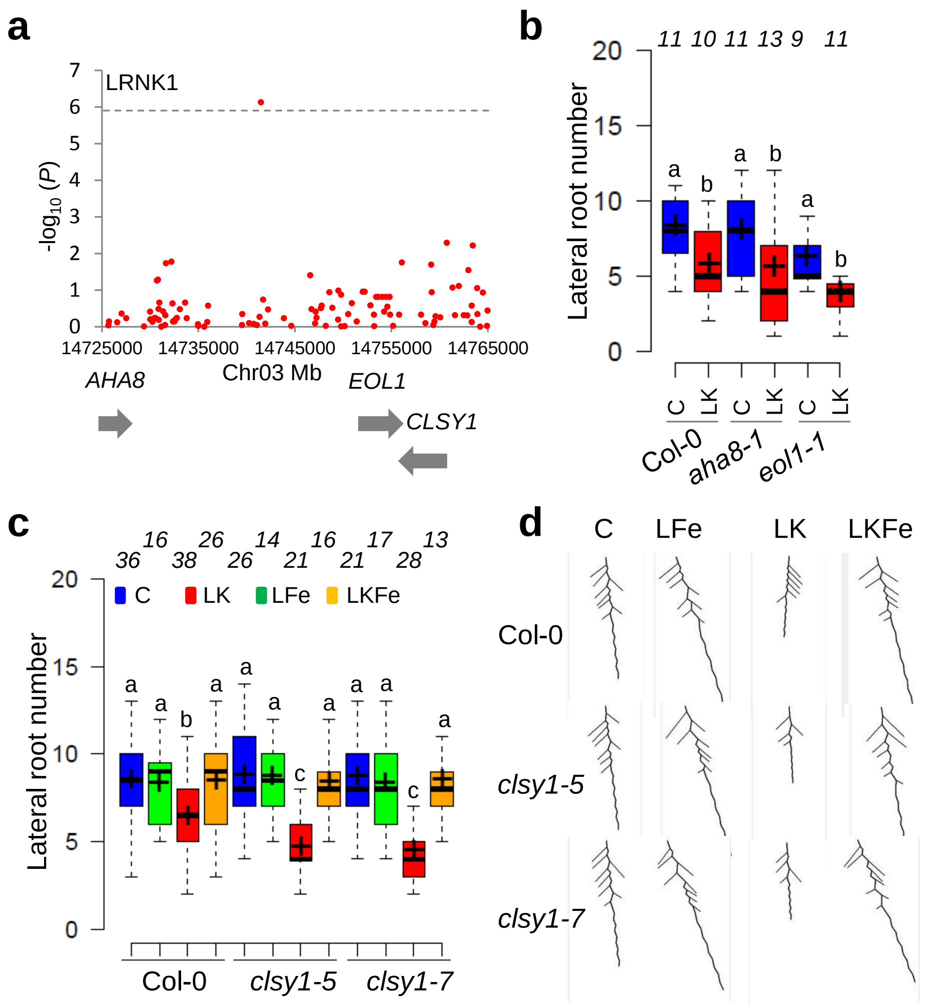

Supplementary Figure 5. **Candidate genes in the vicinity of a significant genome-wide association for lateral root number under low K environment. a.** -log$_{10}$ P values of SNP associations over a 40-kb genomic region surrounding LRNK1. The numbers on the x axis are chromosomal positions in base pairs. The position of gene models of *AHA8*, *EOL1*, and *CLSY1* are indicated below the plot with arrows. **b.** Lateral root number of wild type Col-0, and *aha8-1* and *eol1-1* mutant plants in control (C, blue) and low K (LK, red) environments. The number (n) of plants analysed is indicated above each box in the box plot. Centre lines and crosses in the boxes represent sample medians and means, respectively. Box limits indicate the 25th and 75th percentiles; whiskers extend 1.5 times the interquartile range from the 25th and 75th percentiles. Significant differences between groups at P < 0.05 (one-way ANOVA) are represented by different letters. **c-d.** LR number of of wild type Col-0 and *clsy1* mutant lines in control (C, blue), low Fe (LFe, green), low K (LK, red) and combined low K and low Fe (LKFe, orange). Quantitative data from the indicated number (n) of plants from three independent experiments are shown as box plots (**c**) and visual reconstructions of average root system architecture (**d**). Different letters indicate significant differences at P < 0.001 (one-way ANOVA). The source data of Supplementary Fig. 5b and 5c are provided in a Source Data file.

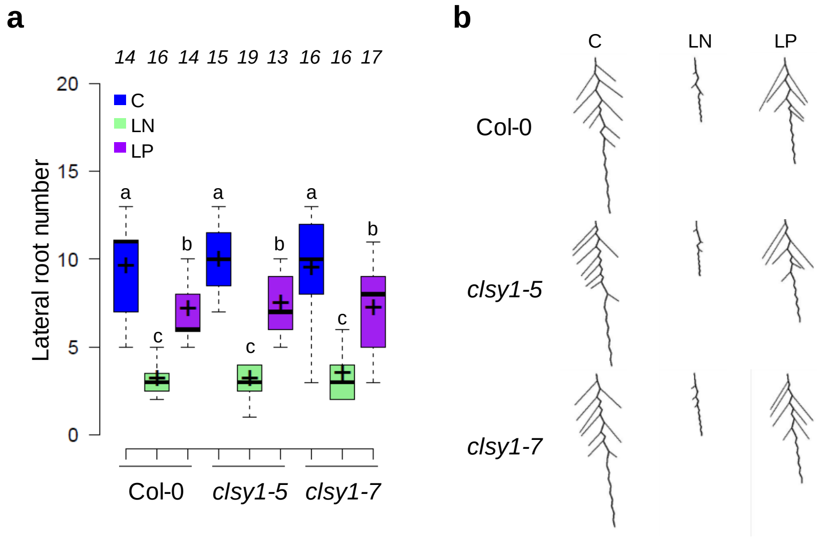

Supplementary Figure 6. ***CLSY1* does not affect *Arabidopsis* lateral root number responses to low nitrogen and low phosphate.** LR: lateral root; LN: low nitrogen; LP: low phosphate. **a.** Box plots of LR number of wild type Col-0 and *clsy1* mutant lines in control (C, blue), LN (light green) and LP (purple). The number (*n*) of plants analysed from three independent experiments is indicated above the boxes in the box plot. Centre lines and crosses in the boxes represent sample medians and means, respectively. Box limits indicate the 25th and 75th percentiles; whiskers extend 1.5 times the interquartile range from the 25th and 75th percentiles. Different letters indicate significant differences at $P < 0.05$ (one-way ANOVA). **b.** Visual reconstructions of average root system architecture of Col-0 and *clsy1* mutant plants in control, low nitrogen and low phosphate conditions are shown. The source data of Supplementary Fig. 6a are provided in a Source Data file.

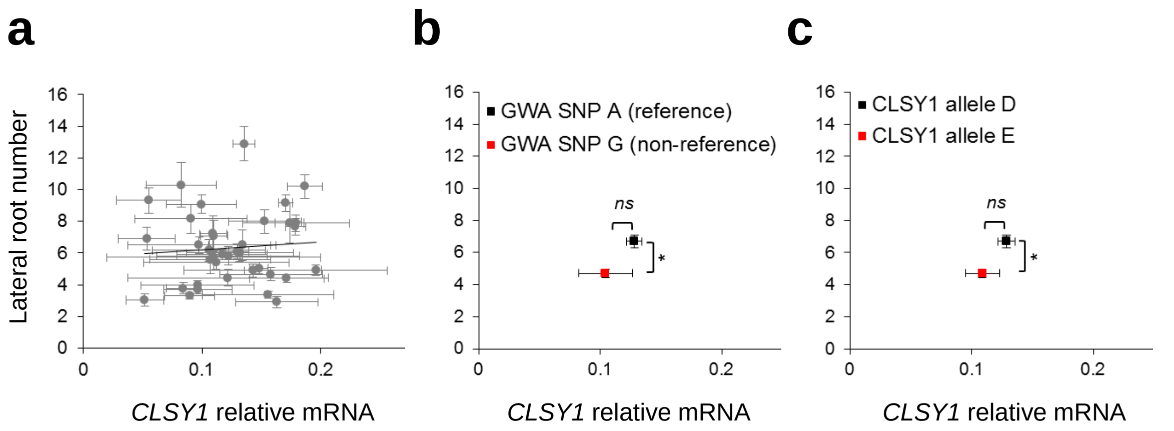

Supplementary Figure 7. **Lateral root number and *CLSY1* mRNA abundance in 37 *Arabidopsis* accessions under low K conditions. a.** For each accession, the mean ± S.E of lateral root number of 12-d-old plants is plotted against *CLSY1* transcript abundance, as determined by qRT-PCR (relative to *PP2A*). Data are from n = 3 independent experiments with roots of 18 plants per experiment pooled for RNA extraction. R is the calculated Pearson correlation coefficient. **b-c.** The same data are shown as means ± S.E of accessions with the GWA SNP (**b**) or CLSY1 allele D/E (**c**) (*ns=non-significant*, * denotes significant differences at $P < 0.05$ (Student's t-test). The source data of Supplementary Fig. 7a-c are provided in a Source Data file.

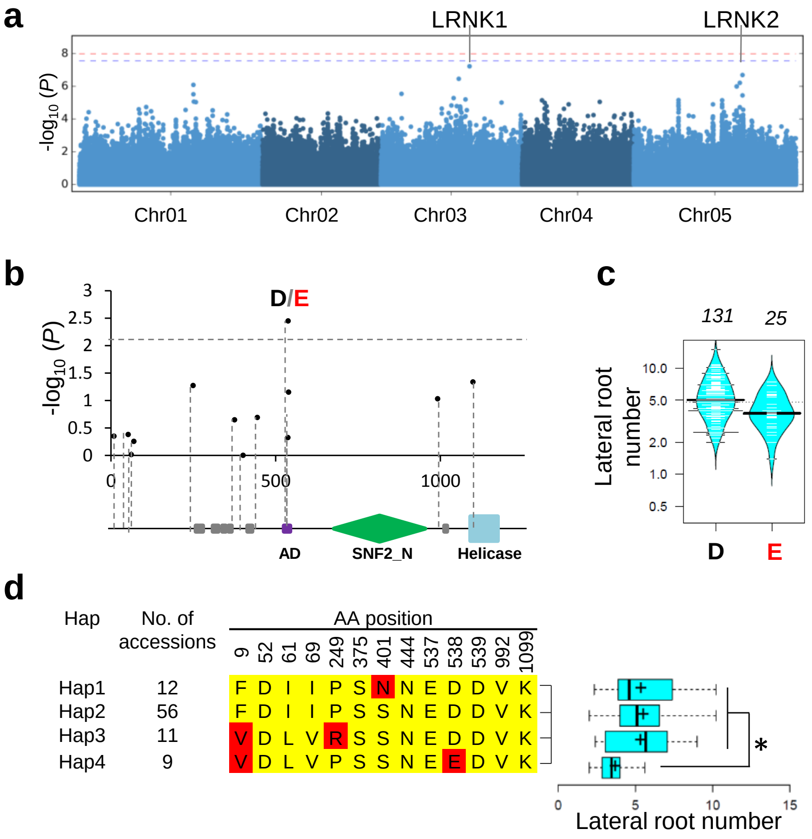

Supplementary Figure 8. Analysis of natural variation of lateral root number under low K conditions in a set of 156 *Arabidopsis* accessions. a. Manhattan plot of GWA mapping for lateral root (LR) number in 156 *Arabidopsis* accessions using Full-seq imputed SNP data and an accelerated mixed-model implemented in GWAPP. A horizontal dashed blue line shows the 5% FDR threshold, and the red line depicts Bonferroni threshold at α = 0.05. LRNK1 and LRNK2 associations previously identified using 250k SNP data for 147 lines (Fig. 1d) are indicated. **b.** Manhattan plot for association with the LR number phenotype of 13 non-synonymous ploymorphisms (MAF > 0.05) within CLSY1 coding sequence in 156 *Arabidopsis* accessions. A general linear model was used for this analysis. A horizontal dashed line shows the 5% FDR threshold. Numbers on *x*-axis are bp starting at the transcription start site. A schematic representation of the predicted domains in CLSY1 protein is shown under the *x*-axis. Low complexity domains, including the acidic domain (AD), were predicted using SMART protein domain annotation resource[2] and are represented by grey and pink boxes, respectively. SNF2_N and Helicase domains are also shown. **c.** Bean plots of LR number of CLSY1 allelic groups, as based on the D538E mutation. Number of accessions belonging to each allelic group is indicated. White lines represent individual data points and centre black lines correspond to the mean values for each group. Estimated density of data for each group are represented by the polygons. Number of accessions carrying D or E CLSY1 allele is shown above the plot. **d.** Haplotypes (Hap) of CLSY1 and LR number phenotype of each haplogroup under low K conditions. Left: The amino acid sequence for each haplotype with variable residues highlighted in red. For this analysis, we only considered the haplotypes with MAF > 0.05 in the 156 accessions panel. Right: Box plot for average LR number measured in each haplogroup. Centre lines and crosses in the boxes represent sample medians and means, respectively. Box limits indicate the 25th and 75th percentiles; whiskers extend 1.5 times the interquartile range from the 25th and 75th percentiles. Asterisk indicates significant difference at *P* < 0.05 (pairwise comparison Student's t-test).

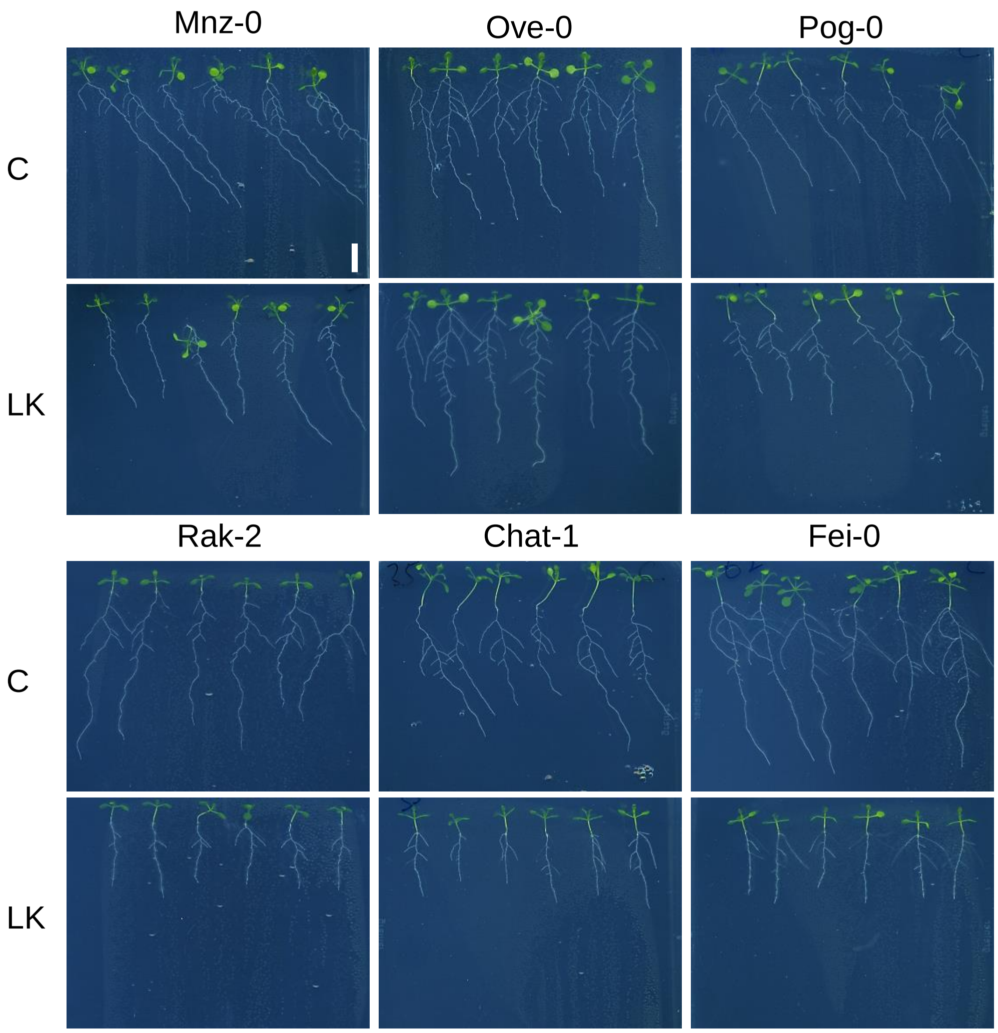

Supplementary Figure 9. **Parental *Arabidopsis* accessions used for quantitative complementation tests.** Representative images of 12-d-old plants of Mnz-0, Pog-0, Ove-0, and Rak-2 accessions harbouring allele D of CLSY1, and of Chat-1 and Fei-0 accessions carrying allele E of CLSY1 under control (C) and low K (LK) conditions. Scale bar is 1 cm.

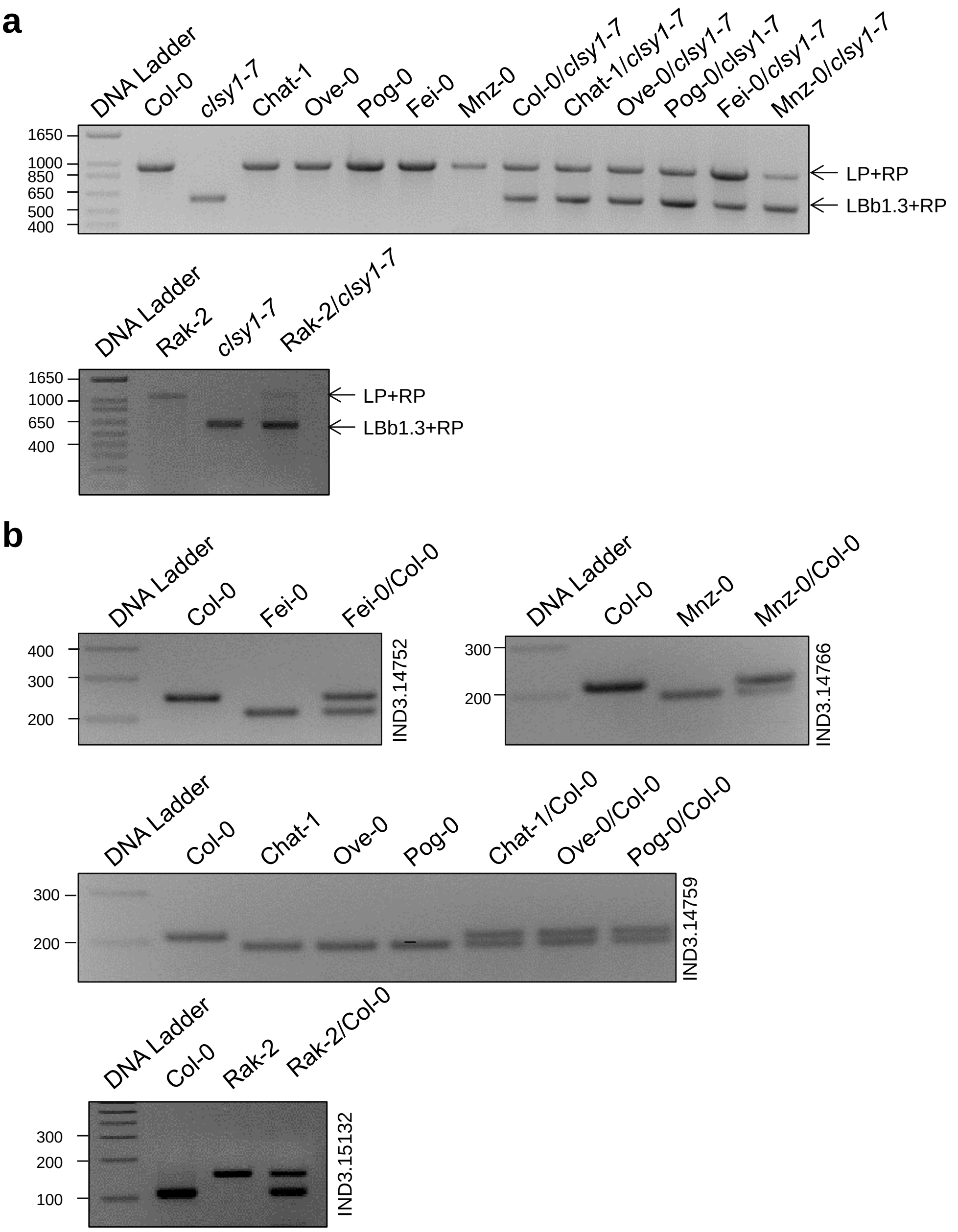

Supplementary Figure 10. **Confirmation of heterozygosity of F1 plants produced by crossing Chat-1, Fei-0, Ove-0, Pog-0, Col-0, Mnz-0 or Rak-2 with either *clsy1-7* or its wild type Col-0. (a)** crossing with either *clsy1-7*; **(b)** crossing with wild type Col-0. Multiplex PCR with primers LP, RP, and with T-DNA left border primer, LBb1.3, was used to amplify wildtype CLSY1 (LP-RP fragment) or CLSY1 with T-DNA insertion (LB1.3 + RP fragment) and was followed by gel electrophoresis to reveal the respective fragment sizes. For crosses with wildtype Col-0 indel markers, IND3.14752, IND3.14766, IND3.14759 and IND3.15132 were used to distinguish each accession. IND3.14759 is located within the *CLSY1* gene, and the markers IND3.14752 and IND3.14766/IND3.15132 are downstream and upstream of *CLSY1* gene, respectively.

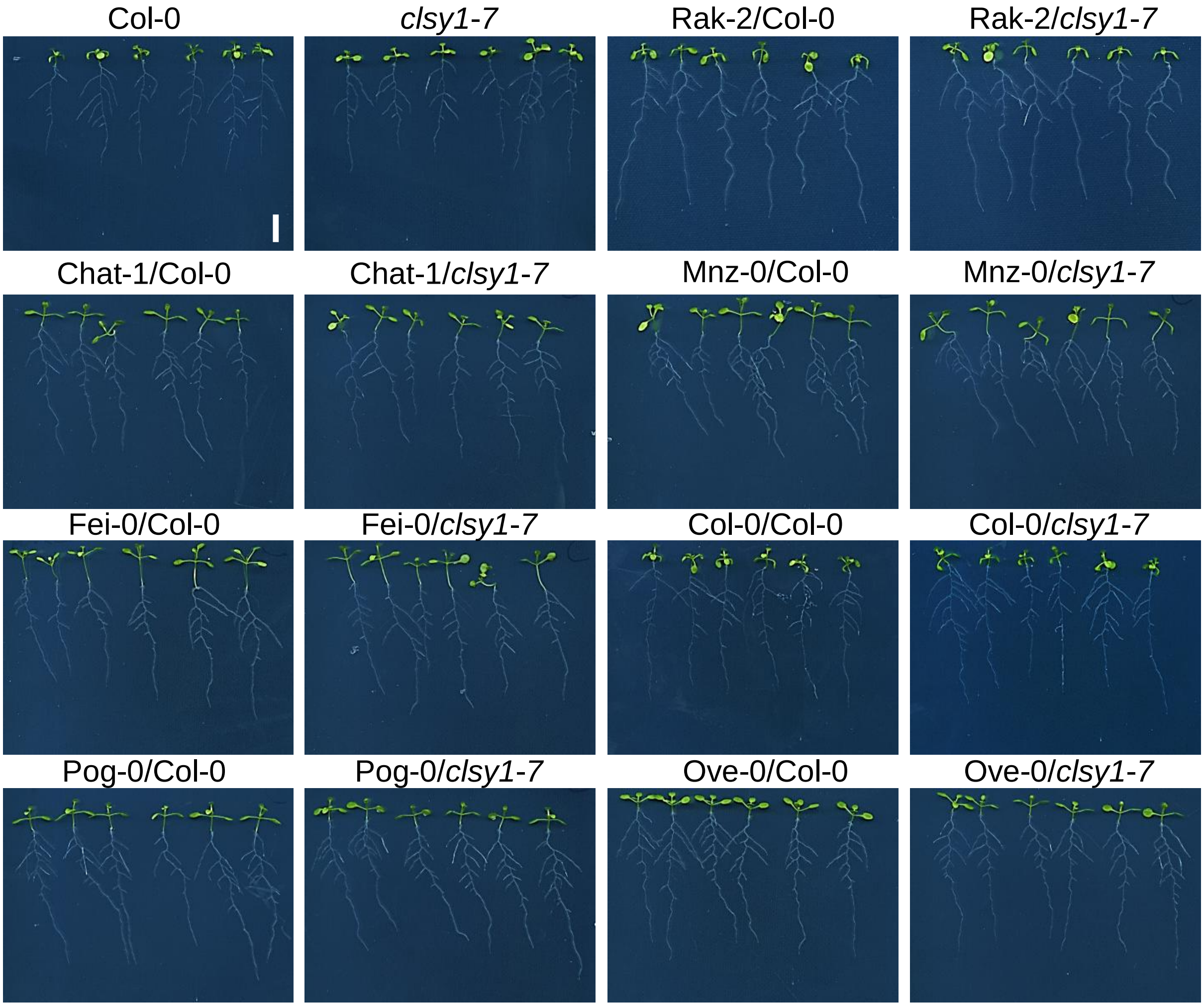

Supplementary Figure 11. **Images of 12-d-old wild type Col-0 and *clsy1-7* mutant plants and of F1 plants originating from crosses of different natural accessions with either Col-0 wildtype or c*lsy1-7*.** Plants were grown in control conditions. Scale bar is 1 cm.

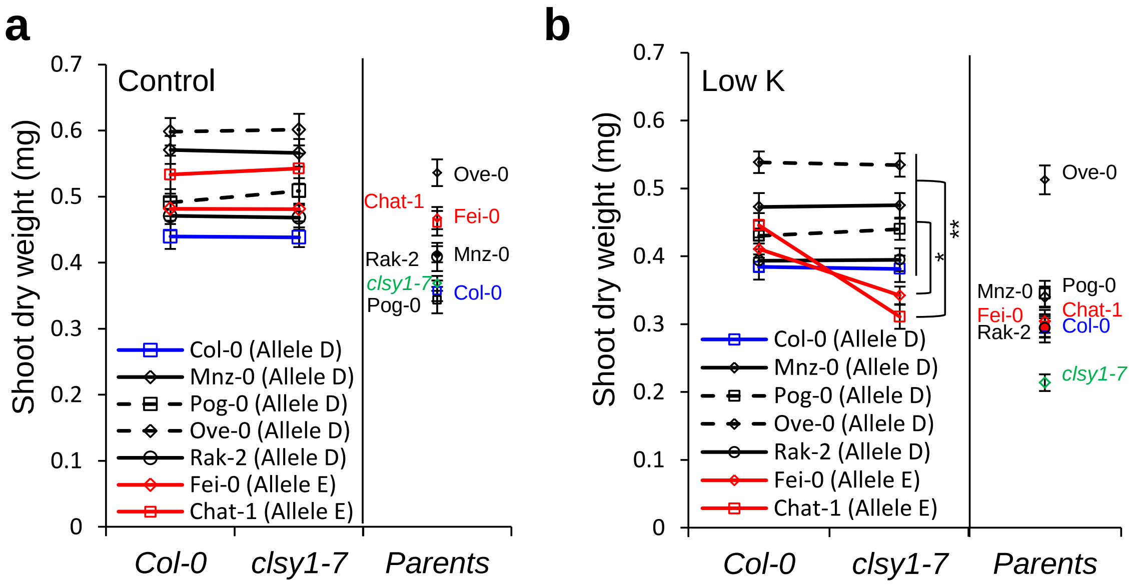

Supplementary Figure 12. Shoot dry weights of different accessions and F1 plants of crosses of *Arabidopsis* accessions with wild type Col-0 or *clsy1-7* mutant plants, in control (a) or low K environments (b). Means ± S.E. of lateral root number from three independent experiments are shown (number of independent plant roots in control/low K *n* = 30/30 (Col-0 x Col-0), 32/32 (Col-0 x *clsy1-7*), 22/35 (Mnz-0 x Col-0), 23/34 (Mnz-0 x *clsy1-7*), 22/37 (Pog-0 x Col-0), 22/34 (Pog-0 x *clsy1-7*), 23/29 (Ove-0 x Col-0), 23/24 (Ove-0 x *clsy1-7*), 27/28 (Rak-2 x Col-0), 28/28 (Rak-2 x *clsy1-7*), 34/39 (Fei-0 x Col-0), 35/37 (Fei-0 x *clsy1-7*), 18/41 (Chat-1 x Col-0), 21/51 (Chat-1 x *clsy1-7*), 27/27 (Col-0 wildtype), 30/34 (*clsy1-7*), 22/24 (Mnz-0), 24/20 (Pog-0), 22/21 (Ove-0), 13/17 (Rak-2), 26/27 (Fei-0), and 26/27 (Chat-1)). The allele x background interactions were tested using Two-way ANOVA, and significant differences are indicated by asterisks (*: $P < 0.05$, **: $P < 0.001$). The source data are provided in a Source Data file.

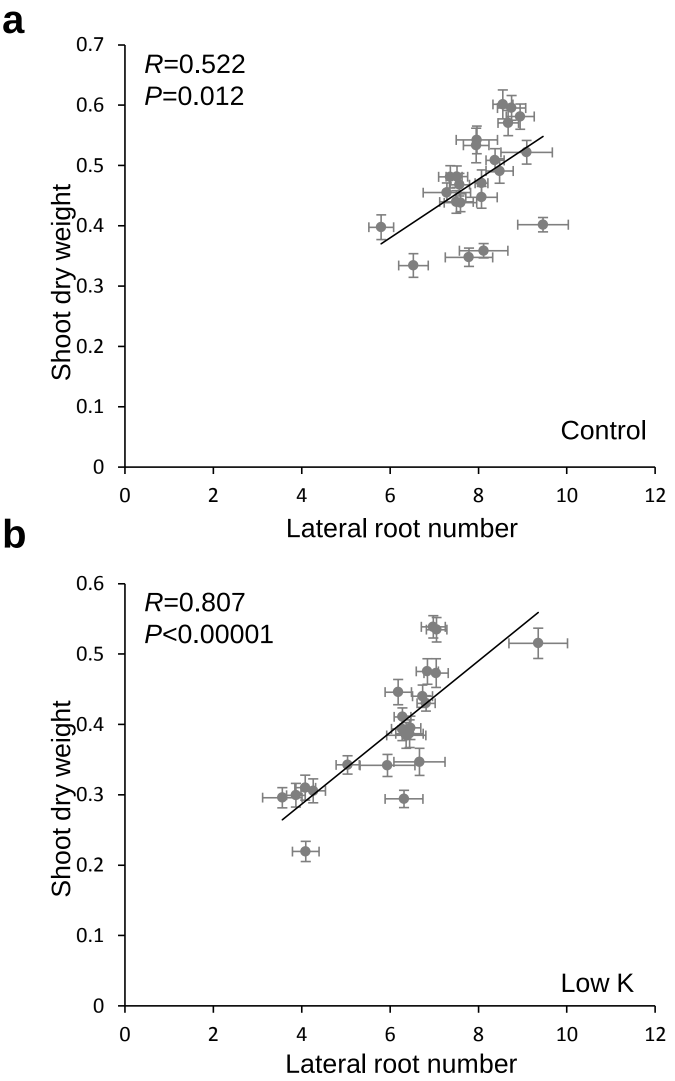

Supplementary Figure 13. **Correlation between lateral root number and shoot dry weight in parental accessions and F1 plants of crosses of accessions with wild type Col-0 or *clsy1-7* mutant plants under control (a) and low K (b) environments.** For each genotype the mean ± S.E of shoot dry weight (DW) is plotted against lateral root (LR) number. Pearson correlation coefficients (*R*) and *P* values indicate strong and significant positive correlation between LR number and shoot DW, particularly in low K. The data are the same as in Figure 2b and Supplementary Figure 11. The source data are provided in a Source Data file.

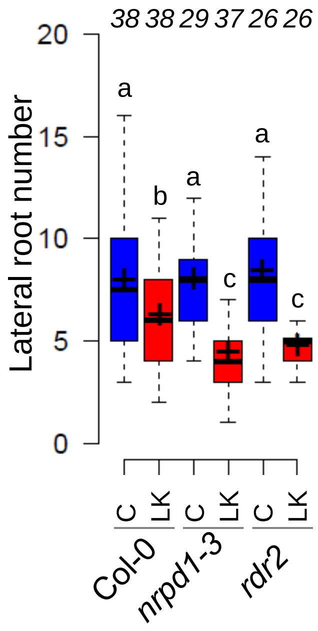

Supplementary Figure 14. Box plots of lateral root number in the indicated number (*n*) of plants from three independent experiments of wildtype Col-0, and *nrpd1-3* and *rdr2* mutants, in control (C) and low K (LK) conditions. Centre lines and crosses in the boxes represent sample medians and means, respectively. Box limits indicate the 25th and 75th percentiles; whiskers extend 1.5 times the interquartile range from the 25th and 75th percentiles. Significant differences between data are represented by different letters ($P < 0.05$ (one-way ANOVA)). The source data of are provided in a Source Data file.

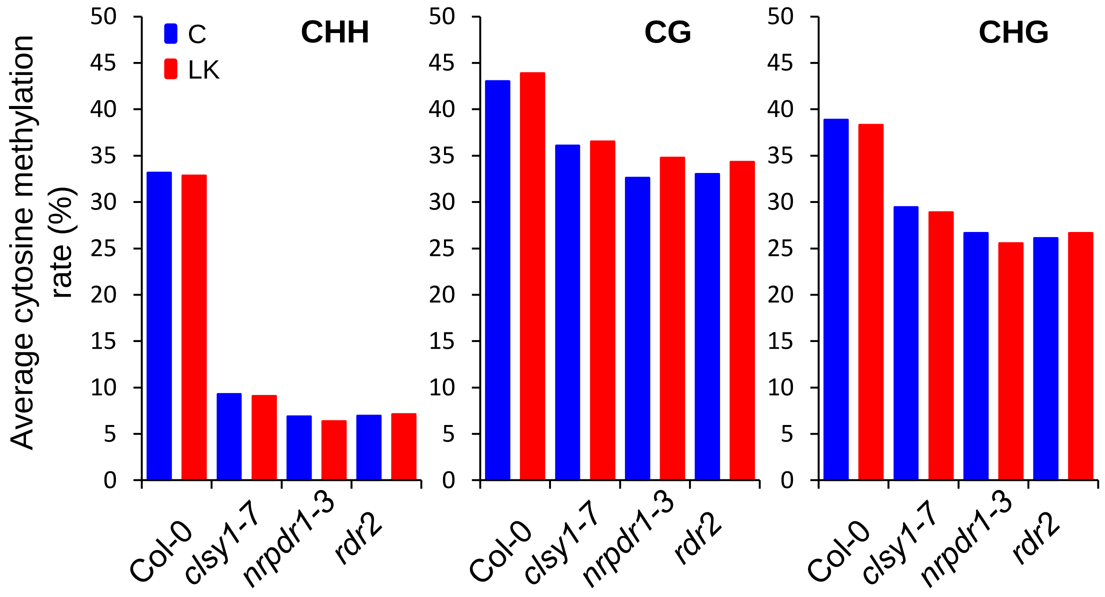

Supplementary Figure 15. **Average DNA methylation rates (%) within a 1.4 kb-promoter region of IAA27 (see Figure 3a).** Plots show methylation of cytosines in the context of CHH, CG, and CHG as determined by DNA bisulfite-sequencing. DNA was isolated from roots of wild type Col-0, and *clsy1-7*, *nrpd1-3*, and *rdr2* plants grown in control and low K environments. The source data are provided in a Source Data file.

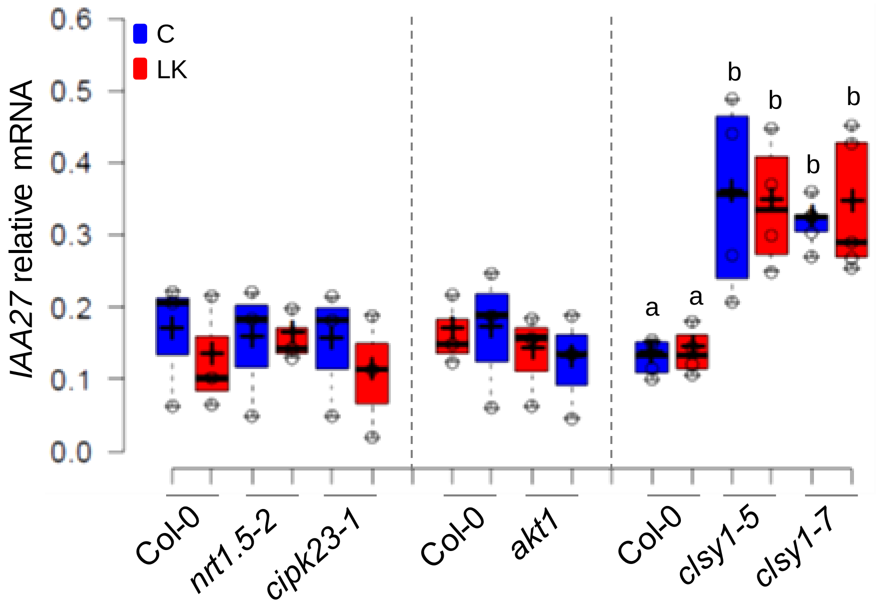

Supplementary Figure 16. **Transcripts levels of *IAA27* in *Arabidopsis* Col-0 wildtype and mutants of *NRT1.5*/*NPF7.3*, *CIPK23*, and *AKT1*. Plants were grown for 12 days under control (C) or low K (LK) conditions.** qRT-PCR was performed to determine the mRNA abundance of IAA27 relative to PP2A. To facilitate comparison, the results for IAA27 mRNA abundance in mutants of *CLSY1* are included in this figure. Data are presented as box plots from *n* = 3 independent experiments. Centre lines and crosses in the boxes represent sample medians and means, respectively. Box limits indicate the 25th and 75th percentiles; whiskers extend 1.5 times the interquartile range from the 25th and 75th percentiles. The source data are provided in a Source Data file.

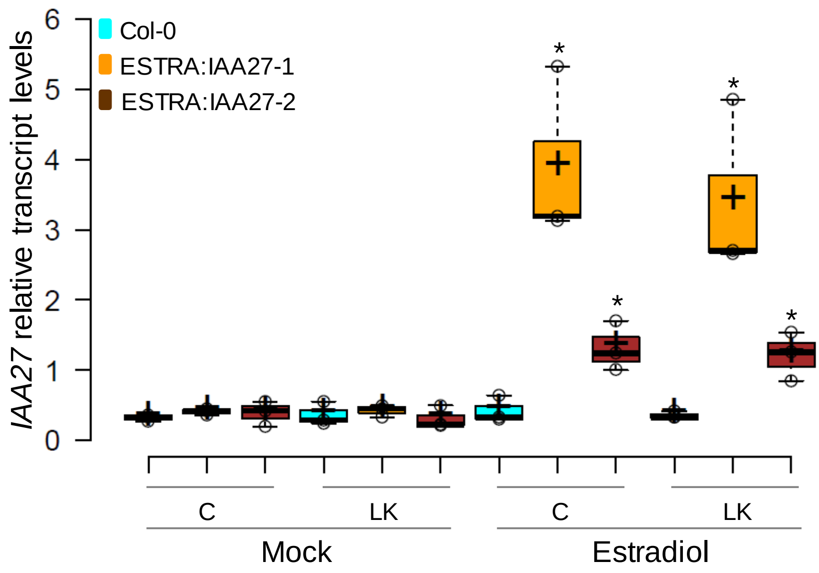

Supplementary Figure 17. **Transcripts levels of *IAA27* in *Arabidopsis* Col-0 wildtype and in transgenic lines (ESTRA:IAA27-1 and ESTRA:IAA27-2) that express IAA27 under the control of an estradiol-inducible promoter**. Plants were grown for 12 days under control (C) or low K (LK) conditions in the absence (mock) or presence of 5 µM β-estradiol. mRNA abundance of IAA27 relative to PP2A was determined by qRT-PCR. Data from $n = 3$ independent experiments are presented as box plots. Centre lines and crosses in the boxes represent sample medians and means, respectively. Box limits indicate the 25th and 75th percentiles; whiskers extend 1.5 times the interquartile range from the 25th and 75th percentiles. Student's t-test was used for pairwise comparisons between mock and β-estradiol treated plants within each condition (asterisks indicate significant differences at $P < 0.05$. The source data are provided in a Source Data file.

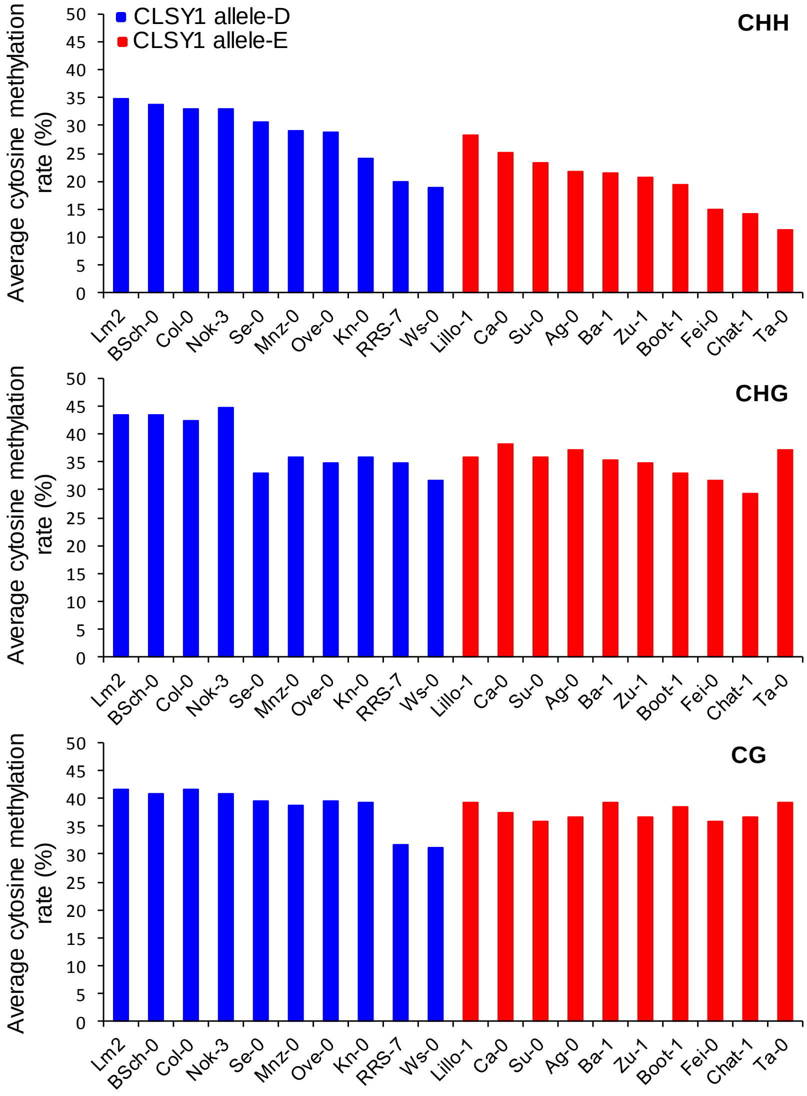

Supplementary Figure 18. **Average rates of DNA methylation (%) within a 1.4 kb-promoter region of *IAA27* (see Figure 3A) in roots of 20 *Arabidopsis* accessions grown in control conditions.** Data for accessions with CLSY1 allele D are shown in blue and data for accessions with CLSY1 allele E are shown by red. Plots show methylation of cytosines in the context of CHH, CG, and CHG. The source data are provided in a Source Data file.

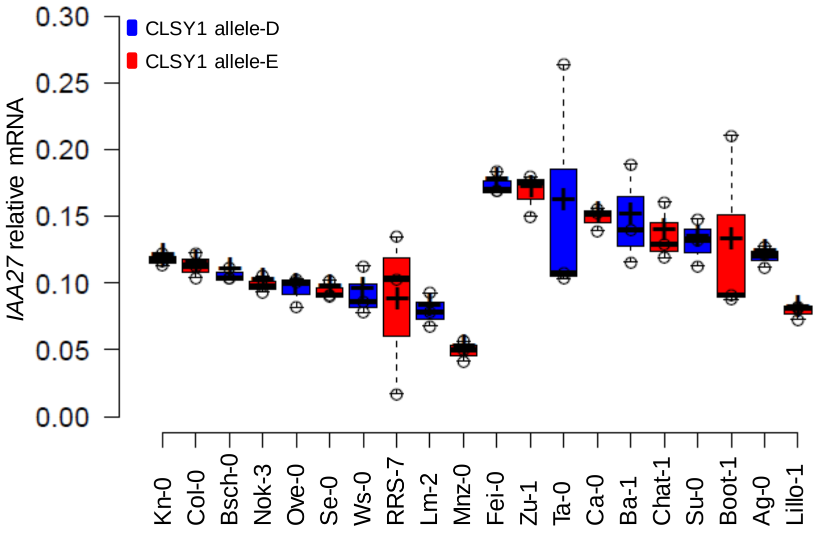

Supplementary Figure 19. **Transcripts levels of *IAA27* in roots of 20 *Arabidopsis* accessions carrying CLSY1 allele D (blue bars) or CLSY1 allele E (red bars).** Plants were grown for 12 days in control conditions. Relative IAA27 mRNA abundance was determine with respect to PP2A by qRT-PCR and data are represented as box plots (n = 3 independent experiments). Centre lines and crosses within box plots represent sample medians and means, respectively. Box limits indicate the 25th and 75th percentiles; whiskers extend 1.5 times the interquartile range from the 25th and 75th percentiles. The source data are provided in a Source Data file.

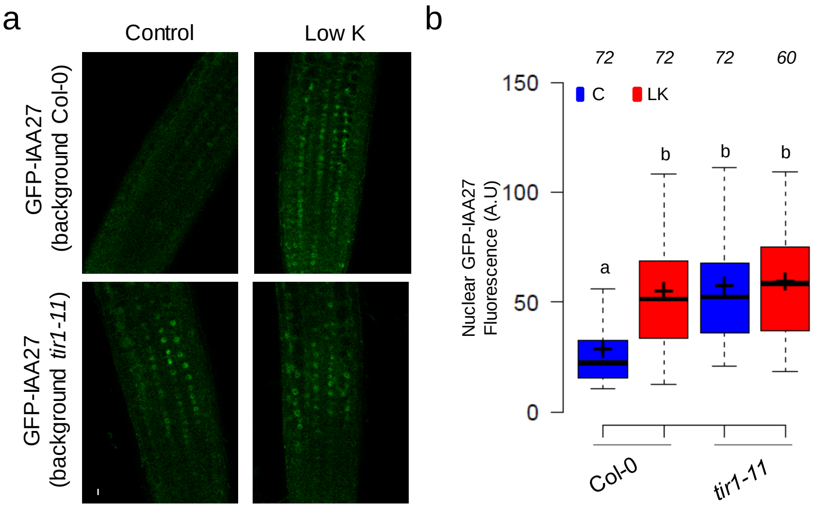

Supplementary Figure 20. **Regulation of prUBQ:GFP-IAA27 fusion protein by K availability and *TIR1* in *Arabidopsis*. a.** Confocal microscopy images of roots of Col-0 and *tir1-11* mutant plants expressing GFP-IAA27 and grown in control or low K environments for 12 days are shown. Scale bar is 10 µm. **b.** Box plots of intensities of fluorescent signal (arbitrary units (A.U.) in indicated number (*n*) of nuclei from 6 plant roots except *tir1-11* in low K where data were obtained from 5 roots. Centre lines and crosses within box plots represent sample medians and means, respectively. Box limits indicate the 25th and 75th percentiles; whiskers extend 1.5 times the interquartile range from the 25th and 75th percentiles. Different letters indicate significant differences at $P < 0.01$ (one-way ANOVA). The source data of Supplementary Fig. 20b are provided in a Source Data file.

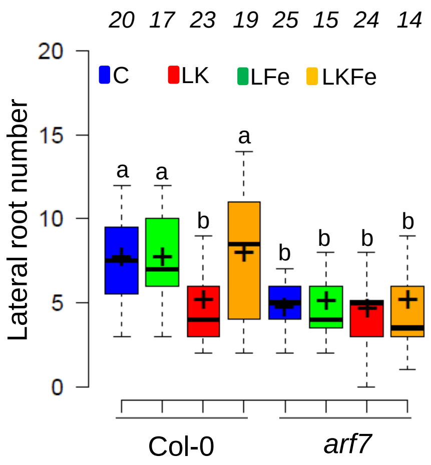

Supplementary Figure 21. Lateral root number in Col-0 and *arf7* mutant plants under control (C, blue), low Fe (LFe, green), low K (LK, red), and combined low K and low Fe (LKFe, orange) conditions. The number (n) of plants phenotyped from three independent experiments are indicated. Centre lines and crosses within box plots represent sample medians and means, respectively. Box limits indicate the 25th and 75th percentiles; whiskers extend 1.5 times the interquartile range from the 25th and 75th percentiles. Different letters indicate significant differences at $P < 0.05$ (one-way ANOVA). The source data are provided in a Source Data file.

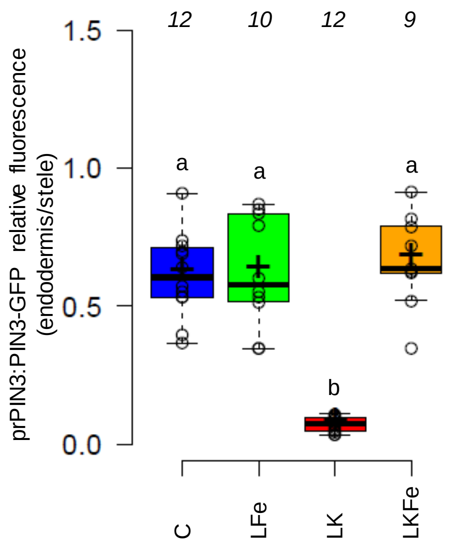

Supplementary Figure 22. Effects of K and/or Fe availability on the expression of prPIN3:PIN3-GFP fusion protein. The box plot shows the fluorescence intensity of PIN3-GFP in the endodermis relative to the stele. Confocal laser scanning microscope was used on 12-d-old roots to visualize PIN3-GFP fluorescence around lateral root initials, and the signal intensity was quantified using ImageJ. Data for indicated number (*n*) of plants from three independent experiments grown in control (C, blue), low K (LK, red), low Fe (LFe, green), and low K and low Fe (LKFe, orange) conditions are shown. Centre lines and crosses within box plots represent sample medians and means, respectively. Box limits indicate the 25th and 75th percentiles; whiskers extend 1.5 times the interquartile range from the 25th and 75th percentiles. Different letters indicate significant differences at $P < 0.001$ (one-way ANOVA). The source data are provided in a Source Data file.

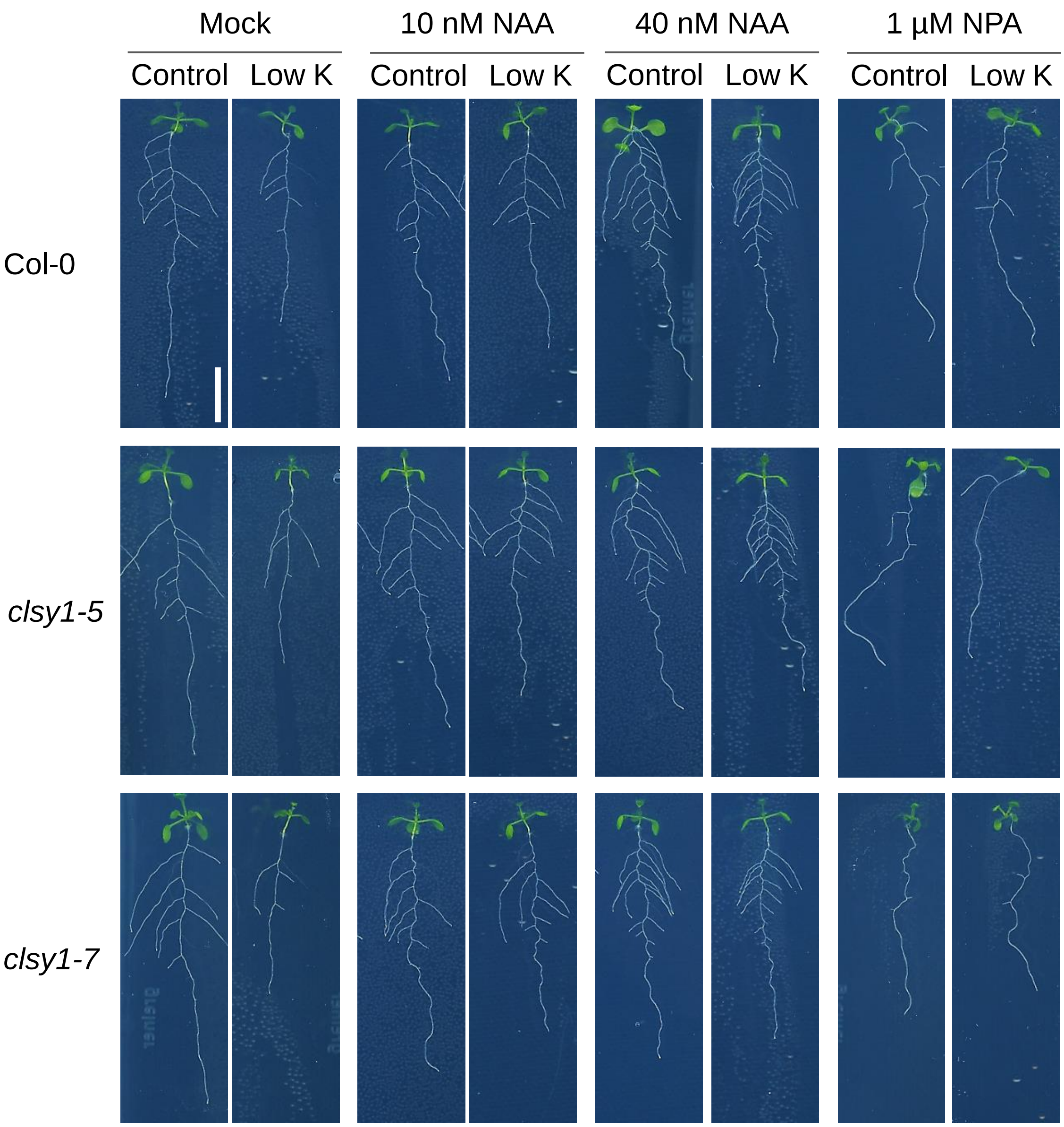

Supplementary Figure 23. **Role of auxin in the lateral root development response to low K availability.** Images of Col-0 wildtype, and of *clsy1-5*, and *clsy1-7* mutant plants are shown. These genotypes were germinated and grown for 12 days under control or low K conditions in the absence or presence of the indicated concentrations of synthetic auxin analogue, NAA, or the auxin transport inhibitor, NPA. Scale bar is 1 cm.

Supplementary References

1. Shahzad, Z. *et al.* EZ-Root-VIS: a software pipeline for the rapid analysis and visual reconstruction of root system architecture. *Plant Physiol.* **177**, 1368–1381 (2018).

2. Letunic, I. & Bork, P. 20 years of the SMART protein domain annotation resource. *Nucleic Acids Res.* **46**, D493–D496 (2018).
