# Supplementary material for: Cryptic variation in RNA-directed DNA-methylation controls lateral root development when auxin signalling is perturbed
Source: Nat Commun. 2020 Jan 10;11:218. doi: 10.1038/s41467-019-13927-3 (PMC6954204; doi:10.1038/s41467-019-13927-3)
Supplement: Supplementary file 3 — Reporting Summary [file 41467_2019_13927_MOESM3_ESM.pdf]

nature research

Corresponding author(s): Anna Amtmann, Zaigham Shahzad

Last updated by author(s): Dec 4, 2019

Reporting Summary

Nature Research wishes to improve the reproducibility of the work that we publish. This form provides structure for consistency and transparency in reporting. For further information on Nature Research policies, see Authors & Referees and the Editorial Policy Checklist.

Statistics

For all statistical analyses, confirm that the following items are present in the figure legend, table legend, main text, or Methods section.

n/a	Confirmed	
☐	☒	The exact sample size (*n*) for each experimental group/condition, given as a discrete number and unit of measurement
☐	☒	A statement on whether measurements were taken from distinct samples or whether the same sample was measured repeatedly
☐	☒	The statistical test(s) used AND whether they are one- or two-sided<br>*Only common tests should be described solely by name; describe more complex techniques in the Methods section.*
☒	☐	A description of all covariates tested
☒	☐	A description of any assumptions or corrections, such as tests of normality and adjustment for multiple comparisons
☐	☒	A full description of the statistical parameters including central tendency (e.g. means) or other basic estimates (e.g. regression coefficient) AND variation (e.g. standard deviation) or associated estimates of uncertainty (e.g. confidence intervals)
☐	☒	For null hypothesis testing, the test statistic (e.g. *F*, *t*, *r*) with confidence intervals, effect sizes, degrees of freedom and *P* value noted<br>*Give P values as exact values whenever suitable.*
☒	☐	For Bayesian analysis, information on the choice of priors and Markov chain Monte Carlo settings
☒	☐	For hierarchical and complex designs, identification of the appropriate level for tests and full reporting of outcomes
☒	☐	Estimates of effect sizes (e.g. Cohen's *d*, Pearson's *r*), indicating how they were calculated

Our web collection on statistics for biologists contains articles on many of the points above.

Software and code

Policy information about availability of computer code

Data collection	Root system architecture data were recorded in EZ-Root-Vis, confocal laser-scanning microscopy was performed using Zeiss LSM510 or Leica SP8 microscope, qRT-PCRs were performed in StepOnePlus RT-PCR system.
Data analysis	Lateral root number data were obtained using EZ-Root-VIS, ImageJ was used to quantify fluorescence intensity, GWAS was done using accelerated mixed models implemented in GWAPP, qRT-PCR data were analysed using StepOnePlus software v2.3, statistical analyses were performed using SigmaPlot 11.0.

For manuscripts utilizing custom algorithms or software that are central to the research but not yet described in published literature, software must be made available to editors/reviewers. We strongly encourage code deposition in a community repository (e.g. GitHub). See the Nature Research guidelines for submitting code & software for further information.

Data

Policy information about availability of data

All manuscripts must include a data availability statement. This statement should provide the following information, where applicable:

- Accession codes, unique identifiers, or web links for publicly available datasets
- A list of figures that have associated raw data
- A description of any restrictions on data availability

The accession numbers are provided in the main article or supplementary section. Data supporting the findings of this work are available within the paper and its Supplementary Information files. Datasets generated during the current study are available from corresponding the authors upon request. The source data underlying Figures 1b, d, 2b, c, 3b, c, 4, 5a, 6b and 7b as well as Supplementary Figures 1b-d, 2b, 3, 5b,c, 6a, 7a-c, 12a,b, 13a,b,14, 15, 16, 17, 18, 19, 20b, 21, and 22 are provided as a Source Data file.

Field-specific reporting

Please select the one below that is the best fit for your research. If you are not sure, read the appropriate sections before making your selection.

☒ Life sciences ☐ Behavioural & social sciences ☐ Ecological, evolutionary & environmental sciences

For a reference copy of the document with all sections, see nature.com/documents/nr-reporting-summary-flat.pdf

Life sciences study design

All studies must disclose on these points even when the disclosure is negative.

Sample size	Precise information on samples sizes is provided for all figures in the Methods section and figures or figure legends. In short, sample sizes vary between 3 and 5 for the analysis of transcript levels (with each sample pooling roots 18-24 plants for RNA extraction), analysis of developmental stages (with each experiment calculating percentage over >40 individual roots) and for the analysis of root architectural feature across all genotypes in the initial GWAS. Average DNA methylation rates and mRNA levels for different alleles were calculated for n = 10 independent accessions representing the alleles. Additional measurements of lateral root number, cell size, dry weight etc. of selected genotypes were carried out and analysed for n > 10 independent individual roots/cells/plants from three independently grown plant batches. Percentage of DNA methylation coverage was calculated from 10 x sequencing.
Data exclusions	No data were excluded from statistical analyses.
Replication	Precise information on replication (number and nature of n) is provided in the Methods and figures/figure legend. See also notes on sample size above.
Randomization	Position of individual plants per genotype/experiment in the growth chamber was randomised and and regularly rotated throughout the growth period.
Blinding	Genome-wide association studies and validation of genes underlying an association were blinded by using codes instead of genotype names.

Reporting for specific materials, systems and methods

We require information from authors about some types of materials, experimental systems and methods used in many studies. Here, indicate whether each material, system or method listed is relevant to your study. If you are not sure if a list item applies to your research, read the appropriate section before selecting a response.

Materials & experimental systems

n/a	Involved in the study	
☒	☐	Antibodies
☒	☐	Eukaryotic cell lines
☒	☐	Palaeontology
☒	☐	Animals and other organisms
☒	☐	Human research participants
☒	☐	Clinical data

Methods

n/a	Involved in the study	
☒	☐	ChIP-seq
☒	☐	Flow cytometry
☒	☐	MRI-based neuroimaging
