# Supplementary material for: Cryptic variation in RNA-directed DNA-methylation controls lateral root development when auxin signalling is perturbed
Source: Nat Commun. 2020 Jan 10;11:218. doi: 10.1038/s41467-019-13927-3 (PMC6954204; doi:10.1038/s41467-019-13927-3)
Supplement: Supplementary file 4 — Description of Additional Supplementary Files [file 41467_2019_13927_MOESM4_ESM.docx]

**Description of Additional Supplementary Files**

File name: Supplementary Data 1
Description: Lateral root number data of 147 *Arabidopsis* accessions under control, low Fe, low K, and low KFe environments.

File name: Supplementary Data 2
Description: Amino acid polymorphisms (MAF>0.05) in the predicted protein sequences of CLSY1 and lateral root (LR) number under low K in 156 *Arabidopsis* accessions.
