# Supplementary material for: Cryptic variation in RNA-directed DNA-methylation controls lateral root development when auxin signalling is perturbed
Source: Nat Commun. 2020 Jan 10;11:218. doi: 10.1038/s41467-019-13927-3 (PMC6954204; doi:10.1038/s41467-019-13927-3)
Supplement: Supplementary file 2 — Peer Review File [file 41467_2019_13927_MOESM2_ESM.pdf]

Reviewers' comments:

Reviewer #1 (Remarks to the Author):

In this work, the authors identify DNA methylation has an important factor in the response of Arabidopsis to K-dependent lateral root development. This result is a very important step in our knowledge of nutrient sensing and lateral root development. This MS, once addressed the comment below, will be suitable for publication.

My major concern is related to the choice made by the authors to present their data. It is very confusing for the reader and should be changed from the beginning to the end.

The story should be simplified. For example: we have done a GWAS on low-K, with identify a SNP (D/E) that explain the K-dependent lateral root density, methylation of IAA27 promoter is affected, then the characterization of the 'auxin gene' towards low-K (tir1, pin3) with DR5-Venus and then what is the specificity of the response (is nitrate-dependent lateral root development also affected?) or low-P and methylation (Secco et al.).

1- Some claims are not supported or could be misleading as "robustness" and "evolutionary innovation":
- Title the use of "robustness" is not appropriate
- Abstract: "Developmental processes strive for a balance between plasticity and robustness to foster evolutionary innovation while maintaining essential functions". I am not convinced that this sentence is of any interest in the abstract. Furthermore, I am not sure it is true.
- we report that genetic variation in RdDM sets the level of robustness ofroot development against environmental or genetic perturbation
2- Figure 1:
a- I do not understand the importance gravistimulation assay since GWAS has been performed on LR number.
b- clsy KO should not be introduced at this point
c- DR5-Venus, we do not understand why this is presented at this point

3- The GWAS should be performed on LR density

4- The genetic analysis combining GWAS and in silico SNP search at the CLSY1 loci is not conventional and not very convincing. It looks like: 'I was very lucky to have a peak near CLSY1 gene and then I am searching for SNP and then make the phenotypic characterization".

5- I do not understand the GWAS on LFe and LKLFe conditions. Why are they presented?

6- other nutrient are known to strongly influence root development, is CLSY1 involved in nitrate-dependent lateral root development

Concerning the missing data:

7- We are waiting a little bit more about the mechanism in action. What is the functional consequence of the mutation? At least in figure 3A had several Arabidopsis ecotypes with different allele. Is the phenotype SCLSY1-specific (what about the other CLSY?)

8- lks mutants have been already characterized (cipk23, npf7.3, other), are they in the same signaling

pathway?

9- sup figure 3 eol1-1 has a phenotype, it is less sensitive to LK

10- “CLSY1 mRNA abundance in the Arabidopsis accessions did not correlate with LR number (Supplementary Fig. 4) indicating that the variation occurs at the protein level”
This is not true

11- what about the natural diversity at the IAA27 allele?

Reviewer #2 (Remarks to the Author):

This well-written manuscript by Shahzad et al., describes that a key component of RNA-directed DNA methylation, CLSY1, safegaurds lateral root development in the face of perturbations in auxin signalling caused by genetic or environmental perturbations. Lateral root development is a key determinant of plant productivity as they largely define the shape of plant root system architecture. Root developmental responses to environmental changes are believed to be adaptive. In this study, authors show that plasticity is not always beneficial for plants, which makes sense. The concept proposed by the authors that DNA methylation serves as a back up for post translational regulations is also novel and well supported by an impressive range of experiments.
While Arabidopsis accessions vary for DNA methylation is reported, this paper clearly explains an important role of this variation to adapt potassium deficient environments.

The experimental designs and data with appropriate statistical analyses shown in the manuscript support the findings. However, there are some points that authors may want to address in the paper.

1. There is a debate about which of the phenotypes, lateral root number or lateral root density, reflects that lateral root development. I understand that in this case lateral root number reflects the lateral root development. Since lateral root development is a rhythmic process, and potassium is known to regulate turgor, could the authors decipher if the inhibition of main root length would be due to a decrease in cell length/size. This could explain why the lateral root number is a good readout for early stages of lateral root development in potassium deficient conditions.
2. The authors found that the non-reference alleles for GWA SNP and CLSY1 associated SNP are in complete LD. Can the authors check and report if this would be true for all the sequenced accessions of Arabidopsis (1001 genome).
3. Out of curiosity, I wonder if there would be any pattern for the distribution of CLSY1 allele D and E across the world. It might be worth testing this again using 1001 genome data.
4. It has been reported that IAA27 interacts with ARF7. The authors should examine the phenotype of arf7 mutant to test how it responds to potassium limitation with respect to the wild type. I believe, this analysis will be essential to further support the model presented in Figure 4E.

Reviewer #3 (Remarks to the Author):

The starting point for this paper is the previous discovery that Arabidopsis roots exhibit increased numbers of lateral roots in response to potassium deprivation. The more detailed analysis of this effect in this paper reveals a decrease in stage II lateral root primordial with an increase in the number of founder cells and stage I primordial. This response was dependent on a high level of Fe and was reversed in low iron. Use of an auxin reporter construct confirmed that the lateral root response was due to perturbation of auxin-regulated processes.
The genetic dissection of this low K response identified two SNPs on chromosomes 3 and 5 and genetic

tests implicated a chromosomal remodeling factor/RNA dependent DNA methylation gene (CLSY1) in this response and with more detailed analysis implicated a non synonymous polymorphism (D to E) in CLSY1 as being responsible for the lateral root phenotype.
The CLSY1 E alleles had a shoot phenotype especially in low K that would be a secondary consequence of reduced lateral root branching.
It is likely that the lateral root phenotype is due to an effect on RNA-directed DNA methylation because other RdDM pathway mutants had a similar phenotype and a CHH hypomethylated (in CLSY1 and RdDM mutants) promoter of an auxin-related gene IAA27 with a corresponding increase in expression. The lateral root phenotypes of the CLSY1 mutant were mimicked by overexpression of IAA27.
The authors elaborate a model that integrates CLSY1-mediated regulation of IAA27 to explain the enhanced lateral root response to low potassium. The model is supported by other data with synthetic auxins and auxin transport inhibitors.
I am quite comfortable with much of the data and some of the interpretation in this paper. The existence of a response to low mineral nutrition is supported by the data and the finding that this response pathway is compromised in an RdDM chromatin mutant is fully justified.
Where I feel less comfortable, however, is with teleology – the attribution of a function – robustness – to the RdDM module in this process. To me it just seems as if the response to low K is reduced in the CLSY1 mutants and it is rather over-egging the custard to call it a manifestation of robustness. I can see how the system allowing the increased branching in low K is a component of the plant's robustness – it allows the plant to continue functioning in response to low K and it contributes a feedback system – low K leads to more lateral roots leads to more K leads to fewer lateral roots and so on. In terms of robustness the important point is the upstream of auxin mechanism that responds to low K – the CLSY1 module is a fine-tuner of the system as evidenced by the increased lateral root formation in the clsy1 mutant although at a lower level than in the wild type.
Apart from this major quibble with the way that the story is framed I think the paper is clear and well written.
There are a couple of typos that could be picked up with spell check:
Undelying
Environemnet
perfirmane
perfirmane
underlies
There are also some technical points that could be spelled out for the general reader including the significance of the founder cells and typeI/II lateral root primordia and more information about the different Arabidopsis panels – Regmap and the extended set of 156 accessions.

Reviewer #4 (Remarks to the Author):

This manuscript by Shahzad et al. titled "Cryptic variation in DNA methylation pathway balances developmental robustness and plasticity" contains characterization of mechanisms of natural variation in development of lateral roots (LRs) under low potassium (K) condition and function of the causal gene in relevance to auxin response. This manuscript highlights interesting approaches towards genome-wide association study and typical genetic analyses using some mutants related to auxin response. They found that one of the component of RdDM (CLSY1) was associated with LR variation in low K condition through the regulation of auxin dependent pathway. Overall, the study appears to be well-performed. However, there are several concern and controversial points as following:
- The authors evaluated the LR variation under the low K condition according to their previous finding of divergent root developmental response to K in Arabidopsis accessions. In addition to that, in this study they tested LR in low and high iron (Fe) condition. Why iron nutrition was particularly considered in LR development with K?
- Genome-wide association mapping detected only two prominent peaks. And the authors described

only LRNK1 in this study. However, LRNK2 also showed significant association as well as LRNK1, or dose the LRNK2 show stronger association than that of LRNK1? Is there epistasis relationship between LRNK2 and 1? How do the authors know that the three candidates within 40 kb around the LRNK1 loci is enough? The significant threshold was not given to other three-condition GWA mapping.

- CLSY1 mRNA variation should be presented between GWAS detected allele. What is the reference allele and non-reference allele in the sentences? It is difficult to read the association allele from the local association study of CLSY1 as shown in Figure S 5b. Linkage disequilibrium can be calculated between CLSY1 locus alleles instead of Table S3.
- Even though there is a variation of LR in both allele, four accession with high LR and two accession with low LR accessions among the variants were selected for evaluation of effect of CLSY1 alleles. Allele effect could be evaluated in random selection according to association alleles or accessions in, for example, Haplotype (4).
- It may explain why shoot growth was inhibited under the low K in connection with LR reduction as shown in clsy1 mutant. Does the LR development affect to take up nutrition?
- CLSY1 (RdDM) regulated methylation on IAA27 RdDM target region under both control and low K condition. There are many reported genes related to usual LR development. It is likely that the RdDM mediates expression of other genes relate to LR development or auxin repose. The author showed the haplotype of CLSY1 associated with LR development, but the methylation on IAA27 target region by CLSY1 (RdDM) was not analyzed in accessions. CLSY1, which was associated with the LR variation, was involved in IAA27-repression by RdDM. However, IAA27 was not detected GWAS at least in LRNK1. This could be represented in variation of IAA27 expression in accessions.
- This study didn’t provide illustration that IAA27 degradation and Aux/IAA and ARF system were suppressed at low K condition inducing lower intracellular auxin, which leads to inhibition of LR development.

Responses to Reviewers:

We are grateful to all reviewers for their constructive comments. We have now carried out a series of additional experiments. Most importantly we have made new crosses to further prove the CLSY1 D/E allele effect, and we have carried out bisulfite sequencing and qPCR for IAA27 in 20 accessions (10 D and 10 E). We have also phenotyped an additional mutant (arf7), and we have generated GFP-IAA27 expressing lines and measured IAA27 protein levels in low K and in *tir1* mutants. The experiments took a considerable amount of time but we are very glad that we did them because they really strengthen our results and conclusions. Thank you for suggesting them.
The first manuscript was in a very short generic format. We have now extended the text according to the Nature Comm format and added figures. This allowed us to clarify and explain better some of the topics raised. We hope that the revised version is makes a good read and satisfies any concerns raised.

Reviewers' comments and responses:

Reviewer #1 (Remarks to the Author):

In this work, the authors identify DNA methylation has an important factor in the response of Arabidopsis to K-dependent lateral root development. This result is a very important step in our knowledge of nutrient sensing and lateral root development. This MS, once addressed the comment below, will be suitable for publication.

My major concern is related to the choice made by the authors to present their data. It is very confusing for the reader and should be changed from the beginning to the end.

The story should be simplified. For example: we have done a GWAS on low-K, with identify a SNP (D/E) that explain the K-dependent lateral root density, methylation of IAA27 promoter is affected, then the characterization of the 'auxin gene' towards low-K (tir1, pin3) with DR5-Venus and then what is the specificity of the response (is nitrate-dependent lateral root development also affected?) or low-P and methylation (Secco et al.).

Response: We appreciate the suggestion by the reviewer to improve the narrative and we have considered it. However, we feel that in the order suggested by the reviewer it will be unclear why we focssed on IAA27 as a potential target of CLSY1. We would therefore like to first characterise the effect of K on LR development at the cellular level including a role of auxin signalling based on the DR5-Venus resuls.

1- Some claims are not supported or could be misleading as "robustness" and "evolutionary innovation":
- Title the use of "robustness" is not appropriate
- Abstract: "Developmental processes strive for a balance between plasticity and robustness to foster evolutionary innovation while maintaining essential functions". I am not convinced that this sentence is of any interest in the abstract. Furthermore, I am not sure it is true.
- we report that genetic variation in RdDM sets the level of robustness ofroot development against environmental or genetic perturbation

Response: We have changed the title and the first sentence of the abstract, but we we think it is important to use the term both in the introduction of the topic and when we interpret our results. . According to a 2015 Nature Rev. Genetics review article "Robustness is characterized by the invariant expression of a phenotype in the face of a genetic and/or environmental perturbation" (Reference 1 in the manuscript). The term is therefore well defined and correctly describes the phenotype of a functional CLSY1: less inhibition of LR (=less phenotypic variance) by low K (=environmental perturbation). Providing a backup circuit that maintains the output when the normal input fails is how enginers introduce robustness into a system, and therefore the term is also appropriate with respect to the molecular mechanism of CLSY1. We have now made an effort to better introduce and discuss the concept of robustness.
Changes: New title. Abstract (l.10-11). Introduction (l. 52-62) Discussion (280-290)

2- Figure 1:
a- I do not understand the importance gravistimulation assay since GWAS has been performed on LR number.
b- clsy KO should not be introduced at this point
c- DR5-Venus, we do not understand why this is presented at this point

Response: a) LR number can be affected by many confounding factors (e.g. main root length, cell size, LR elongation etc.). It is therefore important that we first investigate whether low K has a *bona fide* effect on lateral root development and to characterise it at the cellular level. The gravistimulation assay offers the possibility to trigger LR development at a defined time in a defined location, and therefore allows quantitative comparison of early developmental stages between different treatments and genotypes. b) It is journal policy not to to show the same data twice in different figures. We therefore need to show wildtype and mutant data in the same graph even if the mutant data is explained later in the text. c) The DR-Venus data is part of the initial in-depth characterisation of how low K alters LR development at the cellular level. Less axin/auxin sensitivity under low K motivated the search for auxin-related genes in the set of CLSY1 targets leading sus to IAA27, so it is important to show the DR5-Venus data at the beginning.
Changes: a) We explain the assay in l. 388-391. b) We now explicitely point the reader to the Col-0 data in Figure 1b (l. 94). c) No change.

3- The GWAS should be performed on LR density

Response: We did perform GWAS on over 10 different root architectural features including LR density. The LRNK SNP characterised here was not identified from the GWA analysis for LR density (or any other feature). The reason is very likely that low K also reduces main root (cell) length and therefore, at macroscopic level, lower frequency of LR development is not apparent as a change in LR density. See also Response to Reviwer 2 Comment 1.
Changes: The effect of low K on main root length and cell size is now shown in Supplementary Figures 1 & 2 and described in l. 81-84.

4- The genetic analysis combining GWAS and in silico SNP search at the CLSY1 loci is not conventional and not very convincing. It looks like: 'I was very lucky to have a peak near CLSY1 gene and then I am searching for SNP and then make the phenotypic characterization".

Response: It is well established that SNP patterns that can be very complex in relation to LD,

allele frequency, allelic heterogeneity, population structure and in general the genetic architecture of the trait. Some excellent recent literature (Kerdaffrec, , & Norborg, 2016, *eLife* 5, e22502; Tang, Shahzad, et al., 2016, Nat Comm) explain possible reasons why GWAS may identify some SNPs and not other (linked) SNPs. It is therefore common practice to search for SNPs in nearby genes that could be in LD with the originally identified SNP (see for example JIa et al (2019 Nature Comm. https://doi.org/10.1038/s41467-019-10331-9). Finding them can be considered luck, but this does not make them less meaningful. We prove here quantitatively that the originally identified GWA SNP and the CLSY1 SNP are in complete LD. As in other cases it is possible that allelic heterogeneity masks the latter during GWA mapping.
Changes: We have further extended the SNP analysis to the entire '1001 Genomes' sequence data and still find 100% LD between the GWA SNP and the SNP in the coding region of CLSY1 (main text l. 133-136. Supplementary Table 3).

5- I do not understand the GWAS on LFe and LKLFe conditions. Why are they presented?

Response: The GWAS included these conditions to ensure that any SNPs identified were indeed related to a specific effect of low K with similar dependenc on Fe in other accessions.
Change: We now discuss the Fe-dependency of the low-K phenotype in more detail (l. 273-279)

6- other nutrient are known to strongly influence root development, is CLSY1 involved in nitrate-dependent lateral root development

Response: Several nutrients alter the same macroscopic features of root architecture but the underlying causes and signalling patwhays are not necessarily the same. We have tested *clsy1* mutants under conditions of severe N-deprivation which reduces LR number (moderate N-deficiency does not), and found no phenotype. However, the N-deprived plants are generally very small so it is likely that the difference in LR number is due a more general effect. We also tested low P and again found now phenotype of *clsy1*. In this case it is likely that the lower LR number is a consequence of a strong inhibition of main root length. We do not want to include these data in the paper because it would be important to first exactly describe the effects of other nutrients on LR development at the cellular level and test a variety of different conditions before drawing firm conclusions. This is clearly beyond the scope of this paper.
Changes: No change in manuscript. But see Figure 1 below for your information.

Concerning the missing data:
7- We are waiting a little bit more about the mechanism in action. What is the functional consequence of the mutation? At least in figure 3A had several Arabidopsis ecotypes with different allele. Is the phenotype SCLSY1-specific (what about the other CLSY?)

Response: We have now determined IAA27 DNA-methylations and mRNA levels patterns for 20 accessions (10 with CLSY1 D allele and 10 with E-allel). These results further strengthen our model on the mechanism of CLSY1 function.
We have not tested mutants of other CLASSY genes because they were not identified by the GWAS. Given that they tend to target different genes (Reference 10) it is unlikely that they would show the same phenotype and be additive, but it could be interesting in the future to test whether they have similar functions related to other genes and conditional phenotypes.

Changes: New data on IAA27 DNA-methylation patterns and transcript levels in accessions now shown in Figure 4 an Supplementary Figures 15&16 with text in l. 191-200, and l. 299-312. We have added text on CLSY1 targets and other CLASSY isoforms and references in the Discussion (l. 313-328).

8- lks mutants have been already characterized (cipk23, npf7.3, other), are they in the same signaling pathway?

Response: The specific LR developmental phenotype of clsy1 has not been reported for other mutants and given that CLSY1 itself is not regulated by low K and does not specifically target K-transporters it is unlikely that other l*ks* mutants are in the same pathway as CLSY1. However, the AKT1/CBL/CIPK module is an important part of early perception of low K and could potentially be upstream of the low-K effect.
Changes: We have added some text regarding K-signaling patwhays in the Discussion (l. 267-273)

9- sup figure 3 eol1-1 has a phenotype, it is less sensitive to LK

Response: *eol1*-1constitutively form less LRs. In terms of the response to LK, this mutant shows a comparable response to that of wild type plants.
Changes: No change in manuscript but see Figure 2 below for your information.

10- "CLSY1 mRNA abundance in the Arabidopsis accessions did not correlate with LR number (Supplementary Fig. 4) indicating that the variation occurs at the protein level"
This is not true
Response: Apologies if this sentence was not clear.
Changes: We have changed 'protein'to 'coding sequence' and clarified the text (l. 122-126, l. 128-130). We also now include data on CLSY1 transcript levels separated by allele (Suppementary Figure 5).

11- what about the natural diversity at the IAA27 allele?

Response: No SNP was identified in IAA27. Why natural variation occurs CLSY1 rather than IAA27 itself is an interesting question that cannot be answered here, but we have now included some thoughts in the Discussion as to what could be the advantage of CLSY1 variation.
Changes: Text added in the Discssion (l. 113-128).

Reviewer #2 (Remarks to the Author):
This well-written manuscript by Shahzad et al., describes that a key component of RNA-directed DNA methylation, CLSY1, safegaurds lateral root development in the face of perturbations in auxin signalling caused by genetic or environmental perturbations. Lateral root development is a key determinant of plant productivity as they largely define the shape of plant root system architecture. Root developmental responses to environmental changes are believed to be adaptive. In this study, authors show that plasticity is not always beneficial for plants, which makes sense. The concept proposed by the authors that DNA methylation serves as a back up for post translational regulations is also novel and well supported by an impressive range of experiments.
While Arabidopsis accessions vary for DNA methylation is reported, this paper clearly explains an important role of this variation to adapt potassium deficient environments.

The experimental designs and data with appropriate statistical analyses shown in the manuscript support the findings. However, there are some points that authors may want to address in the paper.

1. There is a debate about which of the phenotypes, lateral root number or lateral root density, reflects that lateral root development. I understand that in this case lateral root number reflects the lateral root development. Since lateral root development is a rhythmic process, and potassium is known to regulate turgor, could the authors decipher if the inhibition of main root length would be due to a decrease in cell length/size. This could explain why the lateral root number is a good readout for early stages of lateral root development in potassium deficient conditions.

Response: Yes indeed; low K shortens main root (cell) length and his could explain why the LR developmental effect described here has no or only a minor effect on LR density.
Changes: We now include data on main root length and cell size in Supplementary figures 1&2.

2. The authors found that the non-reference alleles for GWA SNP and CLSY1 associated SNP are in complete LD. Can the authors check and report if this would be true for all the sequenced accessions of Arabidopsis (1001 genome).

Response: This s a good idea. We now provide data showing that further anlaysis across the accessions from the '1001 genome' confirms that the GWA SNP and the CLSY1 SNP are in complete LD.
Changes: The additional analysis has now been included as Supplemenary Table 3, and is described in the text (l. 133-136)

3. Out of curiosity, I wonder if there would be any pattern for the distribution of CLSY1 allele D and E across the world. It might be worth testing this again using 1001 genome data.

Response: E-alleles seem to cluster on the Iberian peninsula (see Figure 3 below). However, given the fact that the exact origin (e.g. soil properties) is not known for the majority of accessions we hesitate to draw conclusions from this analaysis.
Changes: We have added some thought on evolution in the Discussion (l.334-345).

4. It has been reported that IAA27 interacts with ARF7. The authors should examine the phenotype of arf7 mutant to test how it responds to potassium limitation with respect to the wild type. I believe, this analysis will be essential to further support the model presented in Figure 4E.

Response: Thank you; we have done this now. arf7 mutants phenocopy tir1 and pin3 with respect to K sensitivity of LRN (already low LRN in control, not further descreased in low K), which further supports our model, further confirming that low K acts upstream of the auxin pathway (models now shown in Figs. 6a and 7a)
Changes: The phenotype of arf7 is now included as Supplementary Figure 18 and described in the text (l. 218-222).

Reviewer #3 (Remarks to the Author):

The starting point for this paper is the previous discovery that Arabidopsis roots exhibit increased numbers of lateral roots in response to potassium deprivation. The more detailed analysis of this effect in this paper reveals a decrease in stage II lateral root primordial with an increase in the number of founder cells and stage I primordial. This response was dependent on a high level of Fe and was reversed in low iron. Use of an auxin reporter construct confirmed that the lateral root response was due to perturbation of auxin-regulated processes.
The genetic dissection of this low K response identified two SNPs on chromosomes 3 and 5 and genetic tests implicated a chromosomal remodeling factor/RNA dependent DNA methylation gene (CLSY1) in this response and with more detailed analysis implicated a non synonymous polymorphism (D to E) in CLSY1 as being responsible for the lateral root phenotype.
The CLSY1 E alleles had a shoot phenotype especially in low K that would be a secondary consequence of reduced lateral root branching.
It is likely that the lateral root phenotype is due to an effect on RNA-directed DNA methylation because other RdDM pathway mutants had a similar phenotype and a CHH hypomethylated (in CLSY1 and RdDM mutants) promoter of an auxin-related gene IAA27 with a corresponding increase in expression. The lateral root phenotypes of the CLSY1 mutant were mimicked by overexpression of IAA27.
The authors elaborate a model that integrates CLSY1-mediated regulation of IAA27 to explain the enhanced lateral root response to low potassium. The model is supported by other data with synthetic auxins and auxin transport inhibitors.
I am quite comfortable with much of the data and some of the interpretation in this paper. The existence of a response to low mineral nutrition is supported by the data and the finding that this response pathway is compromised in an RdDM chromatin mutant is fully justified.

Where I feel less comfortable, however, is with teleology – the attribution of a function – robustness – to the RdDM module in this process. To me it just seems as if the response to low K is reduced in the CLSY1 mutants and it is rather over-egging the custard to call it a manifestation of robustness. I can see how the system allowing the increased branching in low K is a component of the plant's robustness – it allows the plant to continue functioning in response to low K and it contributes a feedback system – low K leads to more lateral roots leads to more K leads to fewer lateral roots and so on. In terms of robustness the important point is the upstream of auxin mechanism that responds to low K – the CLSY1 module is a fine-tuner of the system as evidenced by the increased lateral root formation in the clsy1 mutant although at a lower level than in the wild type.

Response: Some statements of the Reviewer are incorrect; e.g. Arabidopsis roots do not 'exhibit increased numbers of lateral roots in response to potassium deprivation' (they show less lateral roots), and there is no 'increased lateral root formation in the clsy1 mutant although at a lower level than in the wild type'. On the contrary, there are less LRs in low K and even less in *clsy1* and the same is true for shoot weight. We would therefore prefer to stick to our interpretation of the results in terms of perturbation and robustness. We have added text to justify our terminology and interpretation. Please see also our response to Reviewer 1 (comment 1) with respect to mechanism.
Changes: Added explanataion and discussion of perturbation/robustness concept. Introduction (l. 52-62) Discussion (280-290)

Apart from this major quibble with the way that the story is framed I think the paper is clear and well written.
There are a couple of typos that could be picked up with spell check:…

Response: Thank you.
Changes: Corrected throughout the text.

There are also some technical points that could be spelled out for the general reader including the significance of the founder cells and typeI/II lateral root primordia and more information about the different Arabidopsis panels – Regmap and the extended set of 156 accessions.

Response: The original manuscript was submitted in a very short generic format. We have now extended the text and provided further explanations. See also Figure 3 below for distribution of the accessions and our response to Reviewer 2 (comment 3).
Changes: Introduction (l. 33-51). Methods (l. 395-411).

Reviewer #4 (Remarks to the Author):

This manuscript by Shahzad et al. titled "Cryptic variation in DNA methylation pathway balances developmental robustness and plasticity" contains characterization of mechanisms of natural variation in development of lateral roots (LRs) under low potassium (K) condition and function of the causal gene in relevance to auxin response. This manuscript highlights interesting approaches towards genome-wide association study and typical genetic analyses using some mutants related to auxin response. They found that one of the component of RdDM (CLSY1) was associated with LR variation in low K condition through the regulation of auxin dependent pathway. Overall, the study appears to be well-performed. However, there are several concern and controversial points as following:

- The authors evaluated the LR variation under the low K condition according to their previous finding of divergent root developmental response to K in Arabidopsis accessions. In addition to that, in this study they tested LR in low and high iron (Fe) condition. Why iron nutrition was particularly considered in LR development with K?

Response: Nutrient interactions have been studied in our lab for a while and it is clear now that they affect root system architecture in many ways (e.g. see Reference 4). An interaction with Fe had already been highlighted for main root inhibition by low P (Reference 37). We therefore included Fe in an initial analysis of root architectural responses to multiple nutrient deficiencies, which was part of a PhD thesis from our lab (Kellermeier PhD), and discovered the KFe interaction. Although we have not yet identified the cause of the interaction it would be wrong not to let the readers know.
Changes: We now include a more detailed discussion of the potential role of Fe in l. 273-279..

- Genome-wide association mapping detected only two prominent peaks. And the authors described only LRNK1 in this study. However, LRNK2 also showed significant association as well as LRNK1, or dose the LRNK2 show stronger association than that of LRNK1? Is there epistasis relationship between LRNK2 and 1? How do the authors know that the three candidates within 40 kb around the LRNK1 loci is enough? The significant threshold was not given to other three-condition GWA mapping.

Response: This paper is about LRNK1. We have not yet characterised LRNK2. We did not pre-set a window size to examine possible causal genes. We simple started with the nearest genes and stopped when we found the phenotype of CLSY1. We used 10% FDR to determine the significance threshold. No SNPs passed this threshold in the three other conditions, therefore we can not determine the significance threshold.
Changes: We have replaced the statement regarding 40 kb with 'nearest genes' and we calrfy that this paper is only about LRNK1 (l. 109-111)

- CLSY1 mRNA variation should be presented between GWAS detected allele. What is the reference allele and non-reference allele in the sentences? It is difficult to read the association allele from the local association study of CLSY1 as shown in Figure S 5b. Linkage disequilibrium can be calculated between CLSY1 locus alleles instead of Table S3.

Responses: Col-0 is the reference allele (A, D) , non-reference is (C, E), see text l. 131, 143. We have now added an analysis of CLSY1 expression by allele and we have carried out additional LD analysis (see also response to Reviewer 2, comment 2).
Changes: Supplementary Fig. 5 now seperates CLSY 1 expression for LRNK1 SNP and for D/E. There was no significant difference but potentially a trend with D-alleles in the upper end of the E-allele range. We now describe these results in the text (l. 123-133). The extended LD analysis is included in Supplementary Table 3.

- Even though there is a variation of LR in both allele, four accession with high LR and two accession with low LR accessions among the variants were selected for evaluation of effect of CLSY1 alleles. Allele effect could be evaluated in random selection according to association alleles or accessions in, for example, Haplotype (4).

Response: The selection of the accessions for the crosses was in fact random, not based on phenotype, but we understand the point that the reviewer is making. We have now generated additional crosses with Rak. Rak2 is Haplotype 1, the only haplotype that was not previously included (the other haplotypes already prove the D/E effect, see Fig. 5e). More importantly, despite the fact that Rak 2 carries a CLSY1 D-allele, it has relatively low LR number. The new results show that Rak2 does complement *clsy1* although the parent phenotype is more similar to the E-allele accessions. This provides strong evidence that complementation of the clsy1 phenotype is due to allele D, not to other factors that contribute to the phenotype.
Changes: The results for Rak2 and Rak2/*clsy1* crosses are now included in Figure 2, Supplementary Figures 8 and 9, and explained in the text (l. 151-154).

It may explain why shoot growth was inhibited under the low K in connection with LR reduction as shown in clsy1 mutant. Does the LR development affect to take up nutrition?

Response: Yes, LR number contributes to total root size (Supplementary Fig. 1) and will therefore generally improve water and nutrient uptake (not just K). A more detailed physiological analysis of the exact relationship between LR number and shoot size is beyond the scope of this study.
Change: We now discuss this in l. 329-335.

- CLSY1 (RdDM) regulated methylation on IAA27 RdDM target region under both control and low K condition. There are many reported genes related to usual LR development. It is likely that the RdDM mediates expression of other genes relate to LR development or auxin repose.

Response: Our search for genes related to auxin signalling and LR development the 3 independent RdDM knockout datasets (References 10, 14, 15) did not reveal any other obvious candidates, although there could of cause be additional genes which have not yet been functionally characterised. There could also be additional conditional phenotypes of CLSY1 linked to other CLSY1 targets that still await discovery.
Changes: Clarifications in text (l. 173) and added discussion (l. 313-328).

The author showed the haplotype of CLSY1 associated with LR development, but the methylation on IAA27 target region by CLSY1 (RdDM) was not analyzed in accessions. CLSY1, which was associated with the LR variation, was involved in IAA27-repression by RdDM. However, IAA27 was not detected GWAS at least in LRNK1. This could be represented in variation of IAA27 expression in accessions.

Response: We have now measured DNA-methylation profiles in the IAA27 target region of 20 accessions (10 D and 10 E) and measured their IAA27 mRNA levels. The results show that D accessions have on average higher CHH methylation levels and and lower expression levels than the E-allele accessions, which provides strong additional evidence for our model (Figure 4). Given that the accessions have different genetic backgrounds it is not surprising that individual values vary between accessions suggesting that other factors contribute to all readouts.
Changes: Added Figure 4, and Supplementary Figures 13 and 14. Results described in text (l. 191-200) and discussed (l. 299-312).

- This study didn't provide illustration that IAA27 degradation and Aux/IAA and ARF system were suppressed at low K condition inducing lower intracellular auxin, which leads to inhibition of LR development.

Response: We already included the results with DR5-VENUS indicating lower auxin levels under low K in LR initials/FC (Fig. 1c and Supplementary Figure 3). As requested by Reviewer 2 (comment 4) we have now added data on *arf7* mutants, which phenocopies *tir1* and *pin3* K (constituively low LR number), thereby strengthening our model. Quantitative measuremet of IAA protein levels *in vivo* is tricky and has not yet been provided by the auxin community. We have now generated Ubq:GFP-IAA27 lines and we can indeed show significantly higher levels of IAA27 protein in low-K treated plants than in control plants and constitutive IAA27 protein levels in *tir1* mutants (Supplementary Figure). These results further strengthen our model. However, the signal/noise ratio is generally low and for quantitative comparison between lines and accessions one would need to develop a more sensitive and ratiometric reporter (and express it in the different genotypes).
Changes: Data for *arf7* mutant is shown in Supplementary Fig. 18 with text in l. 218.
The images and GFP-IAA27 fluorescnece data obtained in roots of control and low-K grown wildtype and tir1 mutants are now included in Supplemengary Figure 17 with text in l. 204-217, and critically discussed in l. 290-298.

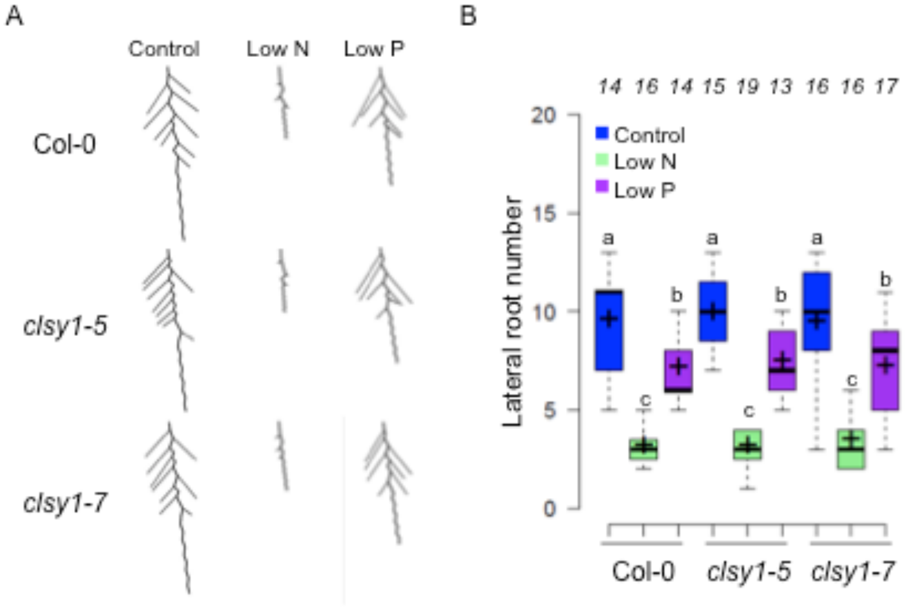

Response figure 1

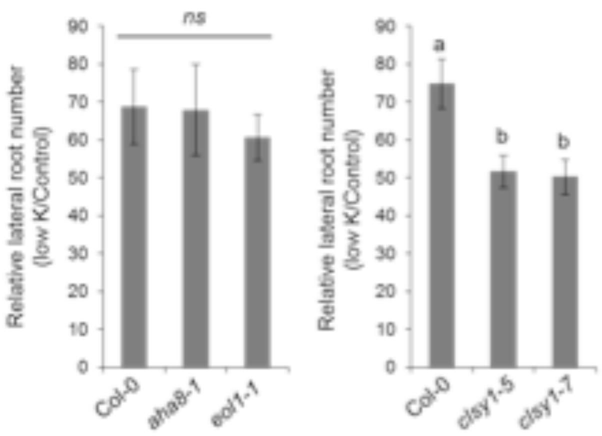

Response figure 2

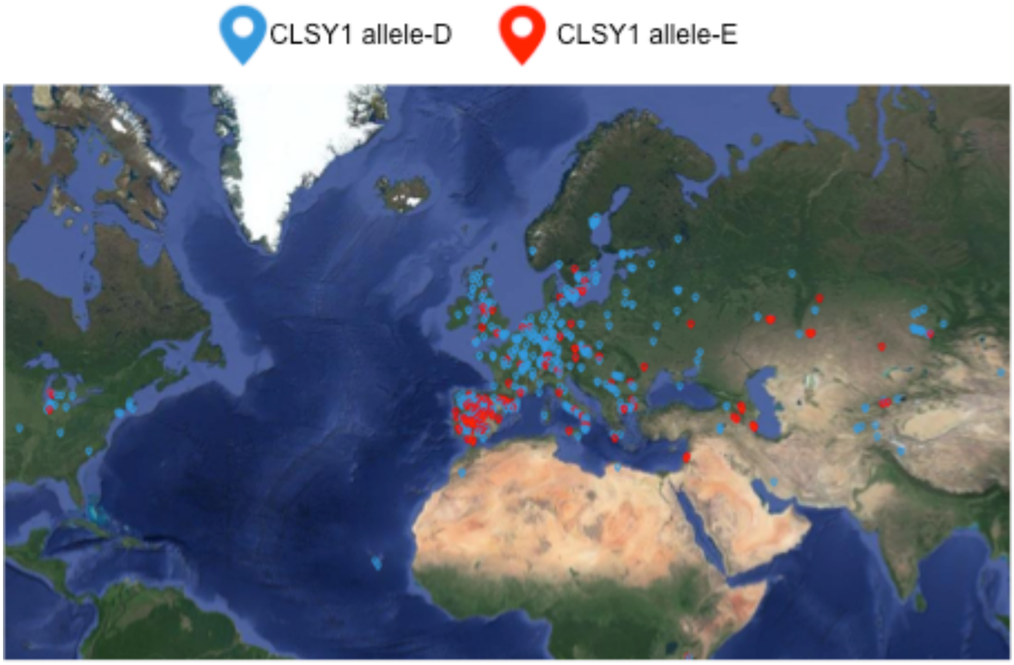

Response figure 3

Reviewers' comments:

Reviewer #1 (Remarks to the Author):

Some data have been added by the authors and this should be acknowledged. I thank the author for these improvments. Some important points have been taken into account, the story is still not clear to me.

Comment 5: I am not convinced

Comment 6: data can be added as supp info

comment 7: what is the functional difference betwwen the two alleles?

comment 8: this is an important point for the specificity of the identified pathwayd (and it is not a difficult and long experiments)

Reviewer #2 (Remarks to the Author):

Authors have fully addressed the reviewer comments. The Reviewer appreciated the new version of the manuscript, including the improved clarity of the text and the new figures.

Reviewer #3 (Remarks to the Author):

First I apologise to the authors for the mistranscription of my notes so that my report referred to increased rather than decreased numbers of lateral roots.

My only defense, that this type of thing is easily done, is illustrated by line 202 of the revised text in which it is stated that "IAA27 over-expression enhance(s) LR development in low K" when the effect is actually a decrease in LR number in low K (Fig 3c).

The authors provide strong evidence that functional consequence of LRNK1 is associated with polymorphism in CLSY1 (through analyses of the D and E alleles in complementation tests with a clsy1 mutant) and its effect on CHH methylation of the IAA27 promoter.

The data also support the complex interaction of the RdDM and auxin pathways as represented by the model shown in Figure 7a and, with the revised manuscript transferring the points about robustness and plasticity to the discussion, I am now content to recommend publication (with correction of the above-mentioned typo).

Reviewer #4 (Remarks to the Author):

This manuscript was well modified with their additional experiments and explanation in the sentences. I don't have further suggestions to this work.

Response to the Reviewers' comments

Reviewer #1 (Remarks to the Author):

Some data have been added by the authors and this should be acknowledged. I thank the author for these improvments. Some important points have been taken into account, the story is still not clear to me.

Comment 5: I am not convinced

Response: As suggested by the reviewer, we have now shifted the Manhattan plots for GWAS in low Fe and low KFe conditions to Supplementary Figure 4. Figure 1d now only shows GWA results for control and low K conditions.

Comment 6: data can be added as supp info

Response: As the reviewer suggests, we have included the results for lateral root responses to low nitrogen and low phosphate as Supplementary Figure 6. A short description of these results is included in the main text (lines 114-118).

Comment 7: what is the functional difference betwwen the two alleles?

Response: This is an interesting follow-on question that needs to be addressed in a comprehensive structure-function study in the future. See our statement in l. 158-160.

Comment 8: this is an important point for the specificity of the identified pathwayd (and it is not a difficult and long experiments)

Response: As suggested by the reviewer we have now tested whether *IAA27* transcription is regulated by *NRT1.5*/*NPF7.3*, *CIPK23* or *AKT1*. IAA27 mRNA abundance was analysed comparatively between Col-0 wildtype plants and mutants under control and low K conditions. We found that none of the mutants had significantly different IAA27 transcript levels compared to wildtype. In particular, they did not show the large increase in IAA27 transcript levels measured in clsy1 mutants (Fig. 3b). As reported before (Fig. 3b) IAA27 transcript levels were also not affected by low-K. The results are now presented as Supplementary Figure 16 and are discussed in the main text (lines 189-191). These new data show that these previously described ‘lks’ mutants (encoding K-transporters or their regulators) do not act through CLSY1. They also further support our initial idea that RdDM is not involved in the perception of the low K signal *per se* (lines 187-189).

Reviewer #2 (Remarks to the Author):

Authors have fully addressed the reviewer comments. The Reviewer appreciated the new version of the manuscript, including the improved clarity of the text and the new figures.

Response: Thank you for your time and constructive input.

Reviewer #3 (Remarks to the Author):

First I apologise to the authors for the mistranscription of my notes so that my report referred to increased rather than decreased numbers of lateral roots.

My only defense, that this type of thing is easily done, is illustrated by line 202 of the revised text in which it is stated that “IAA27 over-expression enhance(s) LR development in low K” when the effect is actually a decrease in LR number in low K (Fig 3c).

The authors provide strong evidence that functional consequence of LRNK1 is associated with polymorphism in CLSY1 (through analyses of the D and E alleles in complementation tests with a clsy1 mutant) and its effect on CHH methylation of the IAA27 promoter.

The data also support the complex interaction of the RdDM and auxin pathways as represented by the model shown in Figure 7a and, with the revised manuscript transferring the points about robustness and plasticity to the discussion, I am now content to recommend publication (with correction of the above-mentioned typo).

Response: Thank you for highlighting this point. We have added precision to this statement (line 211) and similar ones in the text. We also made a few other minor changes in the main text to further improve clarity. These are marked by blue font.

Reviewer #4 (Remarks to the Author):

This manuscript was well modified with their additional experiments and explanation in the sentences. I don’t have further suggestions to this work.

Response: Thank you for your time and constructive input.

REVIEWERS' COMMENTS:

Reviewer #1 (Remarks to the Author):

I would like to thank the authors for their answers. The new data strenghen the hypothesis and the quality of the MS.

REVIEWERS' COMMENTS:

Reviewer #1 (Remarks to the Author):
I would like to thank the authors for their answers. The new data strenghen the hypothesis and the quality of the MS.

Response. We are grateful to the reviewer for their critical input.
